# Homology modeling of major intrinsic proteins in rice, maize and *Arabidopsis*: comparative analysis of transmembrane helix association and aromatic/arginine selectivity filters

**DOI:** 10.1186/1472-6807-7-27

**Published:** 2007-04-19

**Authors:** Anjali Bansal, Ramasubbu Sankararamakrishnan

**Affiliations:** 1 Department of Biological Sciences and Bioengineering, Indian Institute of Technology, Kanpur 208 016, India

## Abstract

**Background:**

The major intrinsic proteins (MIPs) facilitate the transport of water and neutral solutes across the lipid bilayers. Plant MIPs are believed to be important in cell division and expansion and in water transport properties in response to environmental conditions. More than 30 MIP sequences have been identified in *Arabidopsis thaliana*, maize and rice. Plasma membrane intrinsic proteins (PIPs), tonoplast intrinsic proteins (TIPs), Nod26-like intrinsic protein (NIPs) and small and basic intrinsic proteins (SIPs) are subfamilies of plant MIPs. Despite sequence diversity, all the experimentally determined structures belonging to the MIP superfamily have the same "hour-glass" fold.

**Results:**

We have structurally characterized 39 rice and 31 maize MIPs and compared them with that of *Arabidopsis*. Homology models of 105 MIPs from all three plant species were built. Structure-based sequence alignments were generated and the residues in the helix-helix interfaces were analyzed. Small residues (Gly/Ala/Ser/Thr) are found to be highly conserved as a group in the helix-helix interface of MIP structures. Individual families sometimes prefer one or another of the residues from this group. The narrow aromatic/arginine (ar/R) selectivity filter in MIPs has been shown to provide an important constriction for solute permeability. Ar/R regions were analyzed and compared between the three plant species. Seventeen TIP, NIP and SIP members from rice and maize have ar/R signatures that are not found in *Arabidopsis*. A subgroup of rice and maize NIPs has small residues in three of the four positions in the ar/R tetrad, resulting in a wider constriction. These MIP members could transport larger solute molecules.

**Conclusion:**

Small residues are group-conserved in the helix-helix interface of MIP structures and they seem to be important for close helix-helix interactions. Such conservation might help to preserve the hour-glass fold in MIP structures. Analysis and comparison of ar/R selectivity filters suggest that rice and maize MIPs could transport more diverse solutes than *Arabidopsis *MIPs. Thus the MIP members show conservation in helix-helix interfaces and diversity in aromatic/arginine selectivity filters. The former is related to structural stability and the later can be linked to functional diversity.

## Background

Aquaporins and aquaglyceroporins belong to the ancient superfamily of Major Intrinsic Proteins (MIPs) and facilitate passive transport of water and small solutes across membranes of various organisms [1,2]. Aquaporins are likely to have important role in growth, development and stress response in plants [3-5]. MIPs in plants are abundant and constitute a large and highly divergent protein family. For instance, *Arabidopsis thaliana *and *Zea mays *have more than 30 aquaporin-encoding genes each [6,7]. Phylogenetic analysis of plant MIP sequences revealed four major subfamilies: the plasma membrane intrinsic proteins (PIPs), tonoplast intrinsic proteins (TIPs), nodulin 26-like intrinsic proteins (NIPs) and small and basic intrinsic proteins (SIPs). Functionally, two main categories have been well established for MIPs from mammals: water-specific aquaporins and solute-transporting aquaglyceroporins [2]. Most of the plant MIPs that have been investigated are highly specific for water. Several TIPs have been reported to facilitate the transport of urea and ammonia and NIPs have been shown to transport glycerol [8-12]. Recent studies indicate that PIPs contribute to CO2 diffusion across leaf tissues [13]. The substrate specificity of SIP proteins has not been studied in detail.

**Figure 1:**
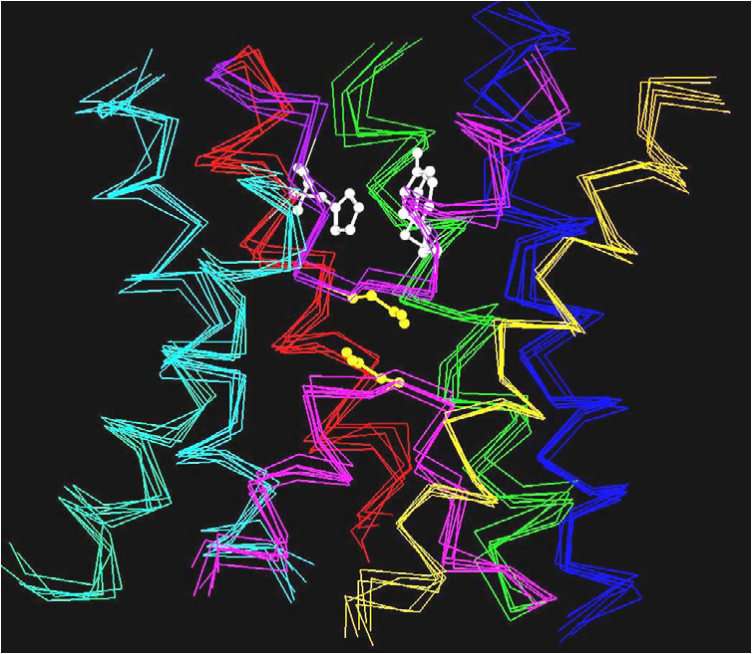
**Superposition of aquaporin crystal structures**. The transmembrane regions of six aquaporin crystal structures (bovine AQP1, *E. coli *AqpZ, sheep AQP0, spinach plasma membrane aquaporin SoPIP2;1, archaeal aquaporin AqpM from *Methanothermobacter marburgensis *and *E. coli *GlpF) are superposed. The corresponding PDB IDs are 1J4N, 1RC2 (B chain), 2B6O, 1Z98 (A chain), 2F2B and 1FX8 respectively. For clarity, Cα traces of only the six transmembrane helices and the loops B and E are shown: TM1 – blue, TM2 – green, loop B – pink, TM3 – orange, TM4 – red, loop E – purple, TM5 – cyan and TM6 – green. The residues forming the Ar/R selectivity filter from SoPIP2;1 are shown in white and the asparagines from the conserved NPA motif of loops B and E are shown in yellow. The aquaporin structures from bacteria, archaea, plant and mammals show a conserved "hour-glass" fold and the helices form a right-handed bundle structure.

The primary sequence of aquaporins exhibits an internal homology and there is ~20% conservation between the N- and the C-terminal halves [14]. The high-resolution structures of several members of MIP family have been determined and are available in the Protein Data Bank (PDB) [15]. Bovine AQP1 [16], GlpF [17] and AqpZ [18] from *E. coli*, sheep and bovine AQP0 [19,20], SoPIP2;1 from spinach [21] and the archaeal aquaporin AqpM from *Methanothermobacter marburgensis *[22], all reveal a homotetrameric organization and each aquaporin monomer forms an independent functional pore. This canonical fold is characterized by a six tilted, membrane-spanning (TM1 to TM6) right-handed helical bundle connected by five loop regions (loops A to E) with N and C-terminal ends located on the cytoplasmic side of the membrane (Figure 1). Loops B and E contain the highly conserved NPA (Asn-Pro-Ala) motifs and form two half-helices that dip into the membrane from opposite sides. The N-terminal ends of these half-helices are connected by interactions between the two NPA boxes and it is also one of the two major constrictions in the channel [1,14]. The second major constriction is located ~8 Å above the NPA region towards the periplasmic side (Figure 1) and this primary selectivity filter known as the aromatic/arginine (ar/R) region is formed by two residues from TM2 and TM5 and two residues from loop E [1,14]. This constriction has been shown as a major check point for solute permeability [23].

**Figure 2:**
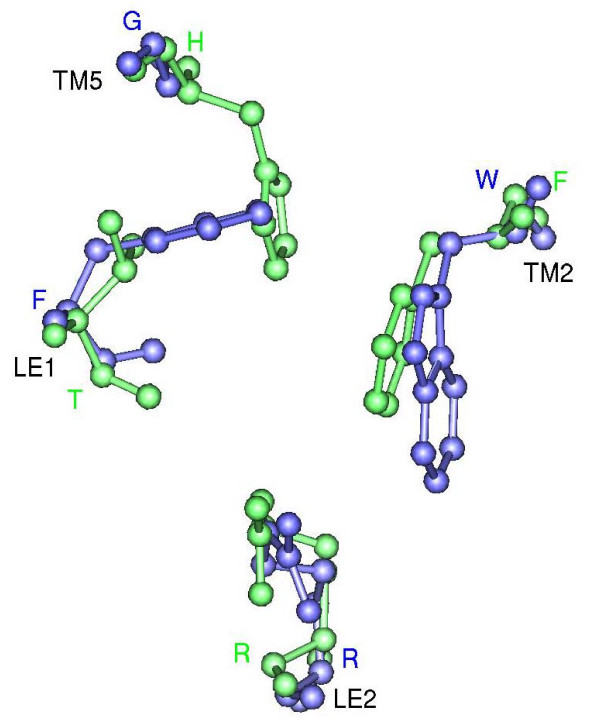
**Ar/R selectivity filters of SoPIP2;1 and GlpF**. Ar/R selectivity filter of water-specific SoPIP2;1 (green) and glycerol specific GlpF (blue). Transmembrane regions of both structures were first superposed and only the residues forming the ar/R tetrad from the superposed structures are shown in ball-and-stick model. Residue names in one letter code are given for SoPIP2;1 in green and for GlpF in blue. The transmembrane segments and the loop regions to which these residues belong are indicated. The projection shown for each filter is viewed perpendicular to the membrane plane from the extracellular side.

The ar/R selectivity filter of spinach plasma membrane intrinsic protein SoPIP2;1 (Figure 2) and three other high-resolution water-specific aquaporin structures, AQP1 [16], AqpZ [18], and AQP0 [19,20], contain a Phe from TM2 (H2 position), His from TM5 (Position H5) and Arg from the loop E (LE2). A fourth residue from loop E (LE1 position) also forms part of the selectivity region by providing its backbone carbonyl oxygen to the ar/R filter and this is usually observed to be a small residue (Cys, Thr or Ala). Crystal structures indicate that His and Arg of ar/R region could provide donor hydrogen bonds for water molecules. The ar/R selectivity filters of *Arabidopsis *PIP members resemble that of water-selective mammalian and microbial aquaporins [24]. In the glycerol-specific GlpF [17], ar/R selectivity filter residues Phe (H2), His (H5) and the smaller residue at LE1 position typically found in water-sepcific channels are substituted by Trp, Gly and Phe respectively (Figure 2). This gives rise to the selectivity filter with larger pore diameter among the known aquaporin structures with two hydrophobic walls opposite the conserved arginine. Such architecture is thought to facilitate the transport of glycerol efficiently [25-28].

Draft genome sequences of rice and more recently, highly accurate finished rice genome sequence have been determined [29-31]. In this paper, we have obtained the complete set of rice aquaporins (OsMIPs) from the rice genomic sequence. We have built three-dimensional structures of rice (OsMIPs) and maize (ZmMIPs) MIPs using homology modeling with AQP1, GlpF and AqpZ crystal structures as templates. Their models were compared with that of *Arabidopsis *(AtMIPs) MIPs. It has been realized that conserved glycines within the transmembrane helices facilitate the closest approaches of helices in the center of the aquaporin helix bundle [18,32,33]. We have generated structure-based sequence alignments in the transmembrane region of all the plant MIPs from the homology models. Conservation of residues in the helix-helix interfaces was analyzed and our results show that residues occurring in the helix-helix interfaces are small and are strongly group-conserved in plant MIPs. Wallace and Roberts [24] have recently performed sequence alignments and homology modeling studies of AtMIPs. Based on the residues that form the ar/R selectivity filter, it was found that TIP, NIP and SIP families diverge from the classical aquaporin structures. Their results suggested that these proteins are likely to have functions distinct from classical aquaporins and aquaglyceroporins. Hence we compared the ar/R selectivity filters of rice and maize with that of *Arabidopsis*. Analysis of pore selectivity regions reveals that NIPs and TIPs from rice and maize are much more diverse compared to AtMIPs. Based on the structural analysis, we have identified potential MIP candidates that could possibly transport diverse solute molecules such as arsenite.

## Results

### Rice MIP sequences

**Figure 3:**
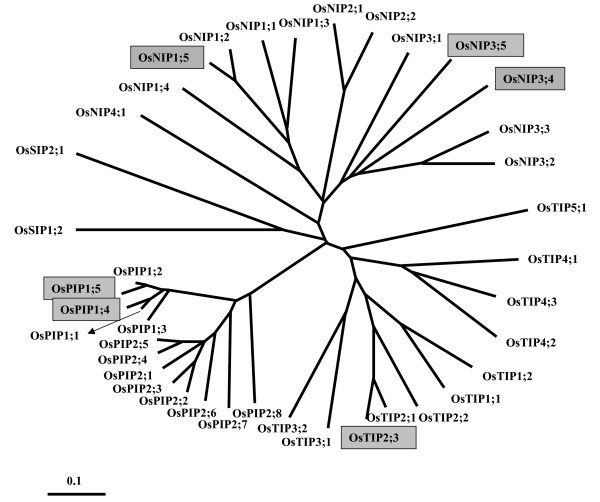
**Additional rice MIPs in the phyologenetic tree of all rice MIPs**. Phylogenetic analysis of all 39 rice MIP sequences is shown. This tree was created using the Neighbor-Joining method and the multiple sequence alignment for this purpose was generated by the T-Coffee program [81]. As observed in *Arabidopsis *and maize, rice MIPs also can be classified into four subfamilies. OsPIPs, OsTIPs, OsNIPs and OsSIPs respectively indicate plasma membrane intrinsic proteins, tonoplast intrinsic proteins, Nod26-like intrinsic proteins and small basic intrinsic proteins from rice. Thirty three out of thirty nine sequences have been identified by Sakurai et al. [43]. The additional six sequences identified in this study are shown within gray boxes.

Identification of rice MIP sequences has become possible due to the availability of complete rice genome sequence. TBLASTN [34-37] searches made in GenBank [38,39], Rice Genome Project (RGP) [40] and The Institute of Genomic Research (TIGR) [41,42] found thirty nine different rice MIP genes. Recently, Sakurai et al. [43] have reported the identification of 33 rice MIP genes and investigated their expression and function. In their study, classification based on phylogenetic analysis indicated the presence of 11 PIPs, 10 TIPs, 10 NIPs and 2 SIPs. Sequence comparisons showed that all the reported 33 MIP genes are identified in our analysis also. We included the six additional MIPs (Table 1) and performed phylogenetic analyses of all 39 proteins (Figure 3). The groups recognized in earlier analyses are more or less intact in the present study. Hence the nomenclature for the 33 sequences is retained as reported by Sakurai et al. [43]. Among the six additional MIPs, there are 2 PIPs, one TIP and 3 NIPs and their names are given based on the subfamily they belong to. The additional PIPs identified in this study are the two mRNA sequences of *indica*-cultivar group available from the GenBank (only these two are from the *indica *subspecies and all other rice MIPs in this study belong to the *japonica *subspecies). OsTIP2;3 has a very long C-terminal extension and hence we have considered only the aquaporin region in this sequence for further analysis. Two of the three NIP sequences have been identified from TIGR release 2 and their amino acid sequences have undergone changes from Release 2 to Release 4. In OsNIP3;4, N-terminus is longer by 43 residues in Release 4. A large deletion is observed in OsNIP1;5 between the two NPA regions. However, prediction of amino acid sequence from genomic sequence using GeneMark [44] supports the sequences from Release 2. Hence in the present study, we have used amino acid sequences from Release 2 for OsNIP1;5 and OsNIP3;4. All the six additional sequences were correctly recognized as MIP/aquaporin sequences in Interpro [45,46] and the rice PIP and TIP sequences identified in this work (OsPIP1;4, OsPIP1;5 and OsTIP2;3) were also found in the database of MIP family of proteins [47,48]. Homology models of 39 rice MIPs, 31 maize MIPs were built as per the modeling protocol described in the Methods section. The same procedure was also used to construct the models of *Arabidopsis *MIPs. In total, 105 models were used to analyze the residue conservation in helix-helix interface and the nature of the ar/R selectivity filters in plant MIPs.

### Residues in the helix-helix interfaces of MIP family members

**Figure 4:**
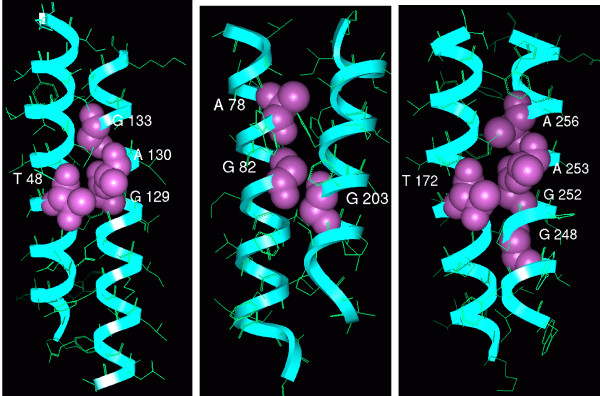
**Small and polar residues at the helix-helix interfaces of SoPIP2;1**. Small and polar residues occurring in helix-helix interfaces of spinach plant aquaporin SoPIP2;1 are displayed. The helix pairs TM1–TM3 (left), TM2–TM5 (middle) and TM4–TM6 (right) are shown. The backbone is drawn in ribbon representation and the interfacial residues are depicted as space-filling models. Residue numbers of interfacial residues correspond to the PDB structure 1Z98.

Table 2 lists the residue pairs occurring at the interfaces of pairs of TM helices or of a TM helix and the half-helix from loops B/E of all aquaporin crystal structures. These residue pairs are within 3.5 Å of the interacting helix in at least one of the template structures used in the modeling study. Such interactions are between either side chain – side chain or side chain – back bone atoms and most are observed between three helix pairs: TM1 and TM3, TM4 and TM6, and, TM2 and TM5 (Figure 4). The sequence motifs GAxxA/GGxxA/GAxxG from TM3, GxxxGAxxA/GxxxGAxxG from TM6 and SxxxG/AxxxG/GxxxG from TM2 have close Cα-Cα contacts (≤ ~6 Å) with helices TM1, TM4 and TM5 respectively. The interface of TM1 and TM2 is also characterized by interactions between a small residue from TM1 (Thr 55 from 1Z98 and equivalent residues from other structures; see Table 2) and the residue that participates in the ar/R selectivity filter from TM2. Additionally, close contacts between the half-helices from loops B and E are observed with TM6 and TM3 respectively. The average distances between Cα atoms of equivalent residue pairs calculated from the six high-resolution crystal structures are also given in Table 2. Most of the distances are less than 6 Å indicating a very close approach of the helix pairs, TM1–TM2, TM1–TM3, TM4–TM6 and TM2–TM5. At least one residue occurring at the helix-helix interfaces can be classified as small and weakly polar residue (Gly, Ala, Ser and Thr). In three cases (TM2–TM5, LB-TM6, LE-TM3), both residues in the interfaces are small. In total, seventeen small and weakly polar residues seem to play an important role in close helix packing in known aquaporin structures (Table 2). We have studied the conservation of these residues in the structure-based sequence alignments of all plant MIPs and also in each of the subfamilies separately.

The occurrence and the role of small and weakly polar residues in helix-helix interfaces of membrane proteins have been extensively investigated in earlier studies [33,49]. Analysis of high-resolution structures of α-helix bundle proteins revealed that high abundance of small residues (Gly, Ser, Thr and Ala) mediate helix-helix interactions in membrane proteins and result in closely packed helices. Hence, for the purpose of this analysis, the residues Gly, Ser, Thr, Ala and Cys have been grouped together and their group conservation is determined in the interfacial positions. We have also reported the individual residues from this group and their conservation if it exceeds 25% (Table 3). All the 17 positions are more than 90% group-conserved in the 105 MIP sequences. Group-conservation for small residues for the helix interfacial positions is the highest (97–100%) for the PIP sub-family followed by the TIP members (94–100%). Although, majority of the small residues are highly conserved in NIP family members, the conservation of Thr 55 and Ala 253 (residues and their corresponding residue numbers reported in this section are from 1Z98 unless otherwise mentioned) falls below 90% in these two positions. Group-conservation of small residues in helix-helix interface is generally high in SIP family, but some positions are poorly conserved (Ala 78, Gly 82 and Ser 181). Many important positions that are likely to affect the interior properties of the channels have been shown to be different in SIP members in comparison with other plant MIP proteins [50]. It should also be noted that the sample size for SIP family is very small (8 members).

While the group-conservation is very strong in all the 17 interfacial positions, there are instances in which subfamilies show strong preference for one or another amino acid of this group (see Additional files 1, 2, 3, 4, 5, 6, 7, 8). For instance, glycine is strongly conserved at positions 82 and 133 in PIPs, but alanine is the preferred amino acid for TIPs in the same position (Table 3). Similarly, alanine is strongly conserved at position 256 in PIPs and TIPs, but glycine has 100% conservation at this position in NIP members.

### Comparison of the Ar/R selectivity filters in rice/maize and Arabidopsis

Wallace and Roberts [24] have recently performed sequence alignments and homology modeling studies of AtMIPs. Based on the residues that form the ar/R selectivity filter, *Arabidopsis *aquaporins have been classified into eight structural subclasses. The amino acid signatures of subclasses belonging to TIP, NIP and SIP families diverge from the classical aquaporin structures and it has been suggested that these proteins are likely to have functions distinct from classical aquaporins and aquaglyceroporins. Ar/R selectivity filter was analyzed in all the homology models generated in this study and compared among the three plant species. All the PIP members from the three plant species have ar/R selectivity filter that shows similarity with water-transporting AQP1. Aromatic/arginine signatures of 30 out of 39 OsMIPs and 23 out of 31 ZmMIPs are identical or similar to their *Arabidopsis *counterparts (Table 4). The characteristics of these selectivity filters have been described in detail in the earlier studies [24] and hence will not be discussed here. MIP members that have unique ar/R selectivity filters found only in rice and maize are discussed in detail below.

**Figure 5:**
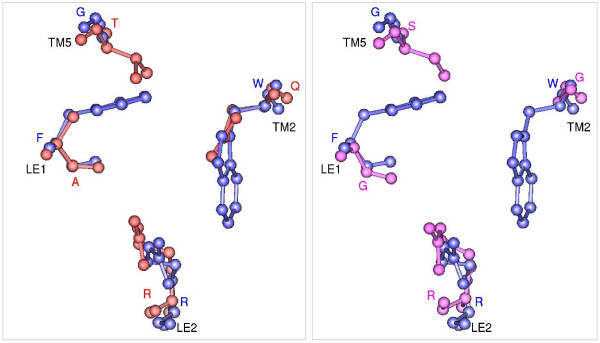
**Ar/R selectivity filters of OsTIP4;2 and OsNIP2;1**. Ar/R selectivity filter of water-specific OsTIP4;2 (red; left) and OsNIP2;1 (pink; right) homology models. Transmembrane regions of both the models were first superposed individually on glycerol transporter GlpF (blue) and only the residues forming the ar/R tetrad from the superposed structures are shown in ball-and-stick model. Residue names in one letter code are given for OsTIP4;2 in red, for OsNIP2;1 in pink and for GlpF in blue. The transmembrane segments and the loop regions to which these residues belong are indicated. The projection shown for each filter is viewed perpendicular to the membrane plane from the extracellular side.

**Figure 6:**
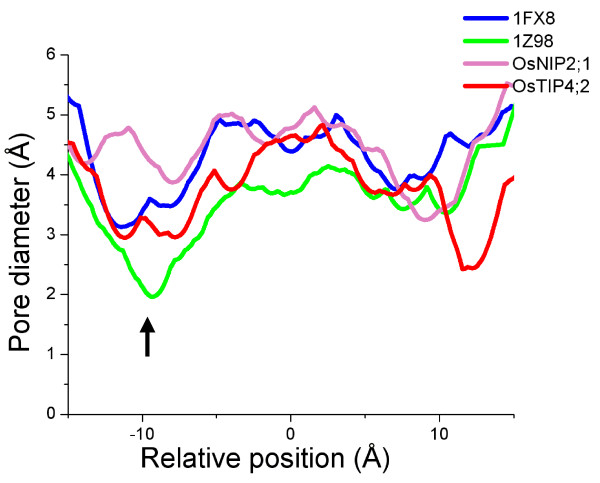
**Comparison of pore radius profiles**. Pore diameter profiles of water-specific SoPIP2;1 (PDB ID: 1Z98), glycerol-specific GlpF (PDB ID: 1FX8), OsTIP4;2 and OsNIP2;1 calculated using HOLE [63]. The black arrow indicates the approximate location of ar/R constriction region. The position Z = 0 Å corresponds to the location of NPA region.

Analysis of homology models shows that some rice and maize TIP family members have ar/R selectivity filters that are not found in *Arabidopsis *and they are listed in Table 5 along with the residues in the four positions that form the ar/R signatures. In five TIPs from maize (ZmTIP4;1, ZmTIP4;2 and ZmTIP4;3) and rice (OsTIP4;1 and OsTIP4;2), the H5 position contains a small hydroxyl residue. In these members, the ar/R selectivity filter is devoid of any hydrophobic residue at both H2 and H5 positions, thus making it highly polar. The LE1 and LE2 positions in this group are occupied by Ala and the highly conserved Arg residue respectively. No *Arabidopsis *TIP gene has an ar/R filter with this feature (Table 5). The selectivity filter of representative member of this group, OsTIP4;2 is shown in Figure 5. The pore diameter at the ar/R selectivity filter region for this model is very similar to that of GlpF (Figure 6), but this constriction is more hydrophilic. In OsTIP4;1, Thr is found at both H2 and H5 positions and as a result this will have a larger pore diameter at the constriction region. In OsTIP5;1 and ZmTIP5;1, the H5 position has Val and hence the selectivity filter is less hydrophilic than the other members from this group.

Small residues (Gly/Ala/Ser/Cys) at H2 and H5 positions are found in seven rice (OsNIP2;1, OsNIP2;2, OsNIP3;2, OsNIP3;5 and OsNIP4;1) and maize (ZmNIP2;1 and ZmNIP2;2) NIP members. Except OsNIP3;5, the position LE1 also possesses a small residue (Gly/Ala) in these members. OsNIP3;5 has a proline in this position. Due to the small size of the residues that form the selectivity filter, these NIP members are likely to have the constriction size that is the largest among all the generated homology models. Residues forming the selectivity filter are shown for a representative member of this group (Figure 5). Due to the small size of the residues, the pore diameter in this region is about 1 Å larger compared to GlpF and about 2.2 Å larger than the water-specific plant PIP structure (Figure 6).

SIP family members are more distantly related to other MIP members and also with the three template sequences. The selectivity filters of SIP members differ significantly from other plant MIP family and any of the known AQP members. For the two *Arabidopsis *members, AtSIP1;1 and AtSIP1;2, the putative ar/R signature obtained in this study (Table 4) differs from the earlier models created by Wallace and Roberts [24]. The H2 position contains a hydrophobic residue (Ile/Val) instead of Thr. Residues in other positions are observed to be the same and hence hydrophobic character of this filter in our model will be greater than that reported in the previous study. In SIP members OsSIP1;1, ZmSIP1;1 and ZmSIP1;2, both H2 and H5 positions are hydrophobic and Pro and Asn are observed in loop E positions.

## Discussion

### Reliability of the plant MIP homology models

**Figure 7:**
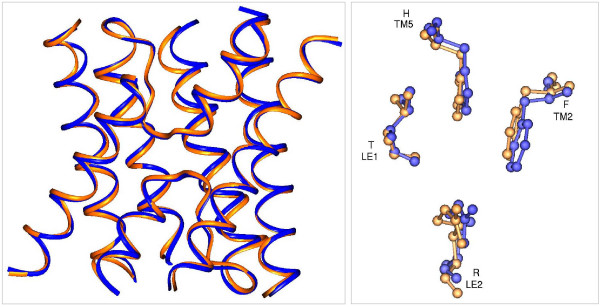
**Comparison of X-ray and model structures of SoPIP2;1**. Homology model generated for SoPIP2;1 is superposed on the experimentally determined structure of SoPIP2;1. Left: Only the transmembrane helical regions and the loops B and E are shown. Right: Residues forming the Ar/R selectivity filters of modeled and the X-ray structures are shown after superposition in ball-and-stick representation. The transmembrane segments and the loop regions to which these residues belong are indicated. There is an excellent agreement between the modeled and the X-ray structures in the transmembrane region.

In this study, we have modeled 105 plant MIP sequences from three different plant species. The initial and an important step in comparative modeling is the selection of the template structure(s). The higher the sequence identity between the sequence(s) of the template structure(s) and the target sequences, the most reliable will be the generated models [51]. In the present study, the sequence identity between the template structures and the target sequences is not very high (26 to 46% for PIPs, TIPs and NIPs; 22 to 29% for SIPs; Table 6). The experimentally determined aquaporin structures from archaea, bacteria, plant and mammals all show a remarkably conserved hour-glass model with right-handed helical bundle structure (Figure 1). For example, the RMSD is less than 1 Å on Cα atoms within the transmembrane regions between the animal and plant aquaporins [21] and the sequence identity between these sequences is ~40%. Based on molecular dynamics simulations, Law and Sansom [52] suggested that homology models based on bacterial homologs may be used to derive meaningful information on the structure, dynamics and function of the corresponding mammalian protein. Before modeling the plant MIPs, we first validated our approach by modeling the SoPIP2;1 structure using the templates from bacteria and mammals and compared with the experimental structure determined at 2.1 Å resolution (Figure 7; see Methods section). The α-carbon backbone of the model generated using three templates shows an excellent agreement with the x-ray structure in the TM region (six TM helices + loops B and E). The higher RMSD observed for the Cα atoms of the whole structure is due to the differences in the loops outside the TM region. Except SIPs, almost all the plant MIP sequences considered for this study have the characteristic sequence features typically found in aquaporin sequences (such as NPA motifs, conserved glutamates in TM1 and TM4 etc.).

Homology models of plant MIPs were characterized and structural subclasses were derived based on the residues forming the ar/R selectivity filter. The ar/R tetrad in PIP, NIP and TIP members from *Arabidopsis *obtained in this study are identical to that reported earlier by Wallace and Roberts [24](see above). They used the MOE homology program [53] based on a segment matching procedure [54]. In the present study, MODELLER [55] was used to build the homology models and SCWRL3 [56] was used to refine the side chains. Although MOE and MODELLER used different modeling strategies, the resultant models from two different approaches have clear agreement in the pore region.

The differences observed in the selectivity filters of one subgroup of SIP sequences in the two studies could be attributed to the fact that SIP sequences are the most diverse sequences. Many structurally important positions in GlpF and AQP1 have been shown to be different in SIPs [50]. For example, the conserved E17 (1J4N numbering) in TM1 is replaced by aspartate. Similarly, F24 in TM1 and Y99 in TM3 are replaced by Trp and Arg respectively. Our modeling approach indeed correctly aligned the residues E17, F24 and Y99 and the highly conserved Q103 in TM3 with the corresponding residues in SIP members (see Additional files 1, 2, 3, 4, 5, 6, 7, 8). However, Wallace and Roberts' method failed to align these positions correctly and several positions in the N-terminal half of SIP proteins are not correctly aligned (see supplementary data of [24]). Hence we believe that our alignment is more accurate and our model which resulted in a different selectivity filter for AtSIPs (Table 4) is more likely to be the correct one. Thus despite low sequence identities with the bacterial and mammalian template structures, the TM region of all the plant MIP homology models gives a reliable starting point to characterize the transmembrane helix packing and pore regions of four MIP subfamilies from three plant species.

### Small residues at helix-helix interfaces are strongly group-conserved in plant MIPs

Analysis of high-resolution structures of 11 membrane and 23 soluble α-helix bundle proteins revealed that high abundance of small residues (Gly, Ser, Thr and Ala) mediate helix-helix interactions in membrane proteins and result in closely packed helices [33]. The high propensity observed for small and weakly polar residues to occur in the closely packed interfaces give rise to motifs such as GxxxG that are known to drive transmembrane helix association [32,57]. Because of the lack of side chain, interfacial Gly residues can participate in weak Cα-H...O type of hydrogen bonds. The small size of the residues can also facilitate inter-helix interactions involving backbone C = O and N-H groups [32]. The functional groups of the Ser, Thr and Cys side-chains can also form inter-helical hydrogen bonds with the backbone C = O and N-H groups across the helix interface. It has been observed that the interfaces of transmembrane helix pairs in GlpF are lined by small and polar residues [33] and networks of Cα-H...O interactions were identified in the high-resolution crystal structure (PDB ID: 1FX8) of this protein [32]. Stroud et al. [18] have suggested that such weak hydrogen bonds could explain the stability of *E. coli *AqpZ in denaturing conditions. Close packing of helices due to the occurrences of small residues at the helix-helix interface has been shown to have functional significance. Recent experimental studies showed that anion permeability of mammalian AQP6 might be due to the substitution of interfacial glycine at TM2 by an Asn residue [58]. This Gly->Asn substitution observed in AQP6 seems to give more flexibility to this protein compared to water-specific AQP1 and such flexibility is proposed to be one of the requirements to convert a water-specific channel to anion permeable channel.

Structure-based sequence alignments have helped in identifying highly conserved active site residues [59,60]. In this study, analysis of sequence conservation in the structure-based sequence alignments of plant MIPs clearly showed that the small residues occurring in the transmembrane helix interfaces are very highly conserved as a group in plant MIPs. This conservation is seen for most of the small residues in the interface even in distantly related SIP families (see Additional files 1, 2, 3, 4, 5, 6, 7, 8). For any one amino acid type, the sequence identity may be 40% (example, Gly 133 position in TM3; Table 3), but when the small and polar amino acids Gly, Ala, Ser, Thr and Cys are considered as a group, the conservation is close to 100%. In the analysis of high-resolution crystal structures, Smith and his coworkers have shown that the molecular notches created by these small residues are a common element of the most closely packed helices in the core of membrane proteins [33,49]. In an analysis of 1047 class A GPCRs, it was revealed that small or polar residues are strongly group-conserved in helix-interface positions [49]. The same analysis was carried out in opsin, amine, olfactory and peptide GPCR subfamilies separately and it was shown that in some positions one or another amino acid of this group is strongly preferred in the subfamilies. In the present analysis of 105 plant MIP sequences, we have shown that the small or polar residues are strongly conserved in the helix-helix interfaces as a group. In the subfamily-specific analysis, we have also observed that in some positions, a specific amino acid of this group is preferred in the subfamilies. For example, position 256 is Ala in PIPs and TIPs and it is Gly in NIPs (Table 3). Similarly in position 55, Thr is predominantly observed in PIPs while most of the other family members have Gly in this position. It should be noted this group conservation at the helix-helix interface would not have been recognized if we had compared just the sequences alone using the conventional multiple sequence alignment tools. In substitution matrices developed exclusively for transmembrane proteins [61,62], the substitution of Thr by Gly or vice versa is more unlikely. This clearly demonstrates the use of structure-based sequence alignment for diverse proteins belonging to the same family where the relationship is difficult to detect.

### Ar/R selectivity filters unique to rice and maize

Nine rice and eight maize MIPs have ar/R signatures that seem to be distinct from any of the *Arabidopsis *MIPs (Table 5). Among the nine OsMIPs, eight have been shown to be expressed [43] and the maize MIPs are derived from the cDNA sequences [7]. The ar/R tetrad of three rice and four maize TIP members (Table 5) is not found in *Arabidopsis*. The conserved feature of five of the members of this group (OsTIP4;1, OsTIP4;2, ZmTIP4;1, ZmTIP4;2 and ZmTIP4;3) is that the residue at H2 position is hydrophilic and the H5 residue is either serine or threonine. This gives rise to a wider hydrophilic constriction. Recent site-directed mutagenesis studies on rat AQP1 showed that replacement of Phe at H2 and His at H5 positions by Ala did not have any effect on water flux [23]. It was concluded that rat AQP1 water permeability is independent of the polarity at the ar/R constriction. However, the increase in constriction diameter did play a role in the mutant rat AQP1, facilitating the transport of bigger molecules like glycerol. Experimental studies have demonstrated that *Arabidopsis *TIP members have been shown to transport urea (AtTIP1;1, AtTIP1;2, AtTIP2;1 and AtTIP4;1) [8] and ammonia (AtTIP2;1 and AtTIP2;3) [10]. To our knowledge, no experimental data for the selectivity of the above rice and maize TIP members is presently available. The hydrophilic putative ar/R selectivity region for this group of TIP members (OsTIP4;1, OsTIP4;2, ZmTIP4;1, ZmTIP4;2 and ZmTIP4;3) with its wider constriction appears to be capable of transporting larger hydrophilic solutes similar to glycerol (Figure 5).

In the ar/R selectivity regions of five OsNIPs and two ZmNIPs (Table 5), all three positions (H2, H5 and LE1) are occupied by small residues, indicating that ar/R constriction of this group will be the largest among all the modeled plant MIPs. Our HOLE [63] analysis shows that the diameter at this constriction is more than 4.0 Å that is ~1.2 Å larger than that of glycerol transporter, the largest known constriction among the experimentally determined structures. Experimental studies have shown that NIP members facilitate the transport of glycerol [12]. It has been speculated that plant aquaporins transport different solutes such as arsenite [64]. Rice and maize NIP members with small residues in three out of four positions of ar/R tetrad (Table 5) have the capability to conduct much larger solute molecules and thus will have distinct structural and functional features, representing a novel group of plant MIPs. This is supported by a recent study that identified a silicon transporter gene in rice [65]. This gene belongs to the aquaporin family and the protein amino acid sequence is identical to that of OsNIP2;1. The same study also suggested that other NIP members, ZmNIP2;1 and ZmNIP2;2, might also be involved in silicon uptake in maize.

A recent study on the two *Arabidopsis *NIP members demonstrated that the residue at H2 position is key in determining the selectivity of the channel [11]. Functional studies showed that AtNIP6;1 with Ala at H2 position exhibited extremely low water permeability but transported larger uncharged solutes like formamide, glycerol and urea. When Ala at H2 was substituted by Trp similar to the ar/R signature of soybean nodulin 26, the archetype of the NIP subfamily, the mutant channel acquired the ability to facilitate water transport and prevented the transport of bulkier urea similar to the soybean nodulin 26 and other NIP members having this signature (Table 4). Due to the presence of a small residue at H2, the pore aperture at the ar/R region increased in NIP6;1. The larger diameter should have resulted in a pore with higher water permeability. Contrary to the expectations, this was not the case. A similar paradox was also observed by Stroud and his coworkers [22]. The ar/R regions in the crystal structures of AQP1, AqpM and GlpF constrict the diameter of the channels to 1.86 Å, 2.54 Å and 3.14 Å respectively. Yet, the most efficient water channel is AQP1 and GlpF is a poor water-conducting glycerol channel. Conductance rate of water in AqpM is relatively low in comparison to AQP1. Thus the cross-sectional surface area of the ar/R selectivity filter and the rate of conductance of water seem to be inversely correlated. A hypothesis based on thermodynamic considerations postulates that in pores with larger diameters, the channel may not be able to properly organize water at the ar/R region [11]. Computational studies have to be carried out to validate this hypothesis. With three of the four in the ar/R tetrad are small residues, in the NIP members like OsNIP2;1 identified in this study, the small residues essentially do not impose any constriction. We propose that these NIP members with diameter larger than that of GlpF in this region are likely to show the rate of water conductance that will be comparable to or even lower than that of GlpF (see above).

SIP subfamily is distantly related in sequence to the other MIP family members and many substitutions in the putative functional regions have been noticed in the analysis of SIP family of proteins [50]. Recent studies using membrane vesicles from yeast cells harbouring one of the *Arabidopsis *SIP members showed that both AtSIP1;1 and AtSIP1;2 have water channel activity [66]; AtSIP2;1 did not show any such activity although the protein was clearly present in the membrane vesicles. The biochemical characterization of the channel and the actual physiological functions remain to be determined for the SIPs. Homology modeling of SIP members shows that the ar/R filters have features that are very different from other MIP members. The conserved Arg at LE2 position is absent in all the SIPs. The challenges imposed due to the sequence divergence resulted in a slightly different model for AtSIP1;1 and AtSIP1;2 in our study (see above). While the model of Wallace and Roberts [24] contained a threonine at H2 position, our model has a hydrophobic residue at the same position. Sequence analysis and putative ar/R signatures suggest that SIPs are most likely to have substrate specificity very different from all known characterized MIPs.

## Conclusion

We have structurally characterized rice aquaporins along with those from maize and *Arabidopsis*. Homology modeling studies were used to build structural models for 105 plant MIPs. Analysis of structure-based sequence alignment of plant MIPs showed that small and weakly polar residues have very high group conservation in the helix-helix interface. We propose that occurrence of small residue in the transmembrane helix interface enables close helix – helix interactions in the transmembrane region in MIP members. Homology models were used to identify the ar/R constriction in all three plant species. Structural characterization based on the ar/R signatures showed that TIP, NIP and SIP members from rice and maize have selectivity filters in the ar/R region that are not found in *Arabidopsis*. A subclass of NIP members has been found to have the constriction with the largest pore diameter since three of the four residues in the ar/R tetrad are small. A recently discovered rice silicon transporter [65] belongs to this group. Members of this subgroup could thus represent a novel group of plant MIPs. SIP members with their unusual ar/R tetrad suggest that their substrate specificity could be very different from known characterized MIP genes. In summary, while the subfamilies diverge in the ar/R signatures that can be directly related to the selectivity of the substrates, a strong conservation of small and polar residues at the helix-helix interfaces indicates that such group conservation is intended to keep the integrity of the "hour-glass" right-handed helical bundle structures in MIP family members. Now that the functional diversity of plant aquaporins has been recognized [67], characterization of novel plant MIPs, identification of new substrates that are transported by these proteins and mechanism of the transport will become the focus of future research that will eventually attempt to answer some of the important questions regarding the role of plant MIPs in root water uptake, reproduction or photosynthesis.

## Methods

### Homology modeling of plant MIPs

Modeling of rice, maize and *Arabidopsis *aquaporins was carried out in two stages. In the first stage, MODELLER [55,68] software package (version 7v7) was used to construct homology models of all the three plant aquaporin proteins. MODELLER derives distance and dihedral angle restraints on the target sequence from its alignment with template 3-D structures and these relationships are expressed as conditional probability density functions. The spatial restraints thus derived and stereochemistry enforced by CHARMM22 force-field terms [69] are combined into an objective function and this function is minimized by an optimization procedure during model building. It has been shown that in MODELLER, using more than one template usually improves the quality of the model [70]. Hence for each of the plant MIP sequence, three high-resolution aquaporin structures [bovine AQP1 (PDB ID: 1J4N; [16], *E. coli *GlpF (PDB ID: 1FX8; [17] and chain B of *E. coli *AQPZ (PDB ID: 1RC2; [18] were used as templates simultaneously in the comparative modeling procedure.

Pairwise sequence alignments between each plant MIP member and the three template sequences were carried out using the 'GAP' program available in the GCG package. Scoring matrix was BLOSUM62 and default values were used for all other parameters. The pairwise sequence identities between members of the MIP subfamilies and the three template sequences (AQP1, GlpF and AQPZ) range from 22.0 to 46% (Table 6). Among the subfamily members, the PIPs are the most closely related to the template sequences and the SIPs are the most distant family members. A multiple structural alignment based on iterative least-squares superposition technique was first carried out on the three template structures. Template sequences thus aligned based on the structural superposition were then used for aligning the target sequence. A dynamic programming method as implemented in MODELLER using "variable gap opening penalty" is used to align the target sequence with the template sequences. This gap penalty avoids placing gaps in secondary structural elements and favours gap in exposed regions and curved parts of the main-chain. Since sequence-structure alignment is a vital step in the model building process, we further checked the target-template alignment manually and gaps in the middle of the helices or in the conserved loops B or E were removed. The knowledge of strictly conserved residues in the transmembrane region reported in the aquaporin sequence analysis studies [71] has been used to further refine the target-template alignment. The residues E17 in TM1, Q103 in TM3, E144 in TM4 and P218 in TM6 (1J4N numbering) are highly conserved and hence alignments in these transmembrane segments have less ambiguity. In a few cases, the alignment was manually adjusted so that the conserved residues in the respective positions are brought under the same column. The default AS1 scoring matrix was used. The resultant alignment was given as input to MODELLER to build models with 'very fast' simulated annealing protocol. For each target sequence, 10 final models were created and the model with the lowest objective function value was selected. The loops of this model were further refined using MODELLER'S loop optimization procedure.

Prediction of side-chain conformation is an important component of the modeling procedure. In the second stage, we have used a side-chain prediction algorithm, SCWRL3 [56,72], on the MODELLER-generated structure. SCWRL3 uses graph theory to solve the combinatorial problem encountered in the side-chain refinement. This algorithm was used to build side-chains of the non-conserved residues on the backbone models generated by MODELLER. Finally, this model was subjected to 200 steps of steepest descent and 200 steps of conjugate gradient energy minimization methods using GROMACS [73,74]. The stereochemical quality of all the models was evaluated using PROCHECK [75,76].

Analysis of the pore dimensions of MIP structural models has been carried out using the program HOLE [63,77]. In this algorithm, a Monte Carlo simulated annealing procedure is used that finds a best path for a sphere with a variable radius to squeeze through the channel. Initially, the model was superposed on the crystal structure of AQP1 (PDB ID: 1J4N) using the "Structure Alignment" option available in the "Homology" module of InsightII (Accelrys, San Diego, CA). This enables easier comparison of pore diameter profiles of different models and hence the coordinates of the superposed structure were used to analyze the pore dimensions. The initial point within the channel is taken as the average of Cα coordinates of the two conserved asparagines (Asn 78 and Asn 194 in 1J4N numbering) and a third residue (Val 178 in 1J4N or an equivalent residue in other models) from TM5 which is located on the channel wall opposite to the conserved asparagines. An initial vector of <0, 0, -1> was specified. AMBER [78]-based van der Waals radius file was used in the calculation of pore dimensions. Crystal structure studies [79] and molecular dynamics simulations [80] suggest that Arg side chain in LE2 position can exist in two distinct conformations, one that maintains a continuous single file of water molecules and the other that completely occludes the channel. It is suggested that alteration between the two conformations can regulate the open probability of the water pore and hence such a fluctuation is proposed to be a good candidate for a possible gating mechanism in aquaporins. In the HOLE calculations, our aim is to find the pore diameter in the open state of the channel. Hence in all the homology models of plant MIPs, the sidechain dihedral angles of Arg in LE2 position were constructed similar to that of water-specific AQP1 channel (PDB ID: 1J4N).

### Validity of the modeling protocol

To test the validity of the approach, we constructed homology models of spinach plasma membrane aquaporin SoPIP2;1. The SoPIP2;1 sequence has 30–44% identity with the three template sequences. A comparison of the experimentally determined structure showed that RMSD of Cα-trace is 4.16 Å and if only the transmembrane helical regions are considered, it is 1.06 Å. In the generated SoPIP2;1 model, a slight improvement in the RMSD of transmembrane helices is seen after side chain refinement and minimization. The superposed experimental and model SoPIP2;1 structures are shown in Figure 7. The helical backbone and the ar/R selectivity filter residues of the model structure are in excellent agreement with the X-ray structure. Small deviations are observed in the termini of transmembrane helices. It has been shown that use of more than one template structure generally increases the accuracy of the models [70]. Hence in the present study, three templates (PDB IDs: 1J4N, 1FX8 and 1RC2-B chain) were chosen for modeling the plant MIPs.

## Authors' contributions

RS conceived and designed the project. AB carried out the project. RS wrote the manuscript. Both the authors approved the final version of the manuscript.

## Supplementary Material

Additional File 1 **Structure-based sequence alignment of plant MIPs in TM1 region**. Structure-based sequence alignments are provided for all the 105 plant MIPs from the three plant species in the TM1 region. The first six sequences correspond to the experimentally determined aquaporin structures from different species. Their respective PDB IDs are shown in the first column. The beginning and end residue numbers are also given for each PDB structure. Small and weakly polar residues (Gly, Ala, Thr, Ser and Cys) occurring in the helix-helix interfaces are shaded in gray color. Click here for file

Additional File 2 **Structure-based sequence alignment of plant MIPs in TM2 region**. Structure-based sequence alignments are provided for all the 105 plant MIPs from the three plant species in the TM2 region. The first six sequences correspond to the experimentally determined aquaporin structures from different species. Their respective PDB IDs are shown in the first column. The beginning and end residue numbers are also given for each PDB structure. Small and weakly polar residues (Gly, Ala, Thr, Ser and Cys) occurring in the helix-helix interfaces are shaded in gray color. The residues forming arginine/aromatic selectivity filter are shown in bold. Click here for file

Additional File 3 **Structure-based sequence alignment of plant MIPs in TM3 region**. Structure-based sequence alignments are provided for all the 105 plant MIPs from the three plant species in the TM3 region. The first six sequences correspond to the experimentally determined aquaporin structures from different species. Their respective PDB IDs are shown in the first column. The beginning and end residue numbers are also given for each PDB structure. Small and weakly polar residues (Gly, Ala, Thr, Ser and Cys) occurring in the helix-helix interfaces are shaded in gray color. Click here for file

Additional File 4 **Structure-based sequence alignment of plant MIPs in TM4 region**. Structure-based sequence alignments are provided for all the 105 plant MIPs from the three plant species in the TM4 region. The first six sequences correspond to the experimentally determined aquaporin structures from different species. Their respective PDB IDs are shown in the first column. The beginning and end residue numbers are also given for each PDB structure. Small and weakly polar residues (Gly, Ala, Thr, Ser and Cys) occurring in the helix-helix interfaces are shaded in gray color. Click here for file

Additional File 5 **Structure-based sequence alignment of plant MIPs in TM5 region**. Structure-based sequence alignments are provided for all the 105 plant MIPs from the three plant species in the TM5 region. The first six sequences correspond to the experimentally determined aquaporin structures from different species. Their respective PDB IDs are shown in the first column. The beginning and end residue numbers are also given for each PDB structure. Small and weakly polar residues (Gly, Ala, Thr, Ser and Cys) occurring in the helix-helix interfaces are shaded in gray color. The residues forming arginine/aromatic selectivity filter are shown in bold. Click here for file

Additional File 6 **Structure-based sequence alignment of plant MIPs in TM6 region**. Structure-based sequence alignments are provided for all the 105 plant MIPs from the three plant species in the TM6 region. The first six sequences correspond to the experimentally determined aquaporin structures from different species. Their respective PDB IDs are shown in the first column. The beginning and end residue numbers are also given for each PDB structure. Small and weakly polar residues (Gly, Ala, Thr, Ser and Cys) occurring in the helix-helix interfaces are shaded in gray color. Click here for file

Additional File 7 **Structure-based sequence alignment of plant MIPs in the loop B region**. Structure-based sequence alignments are provided for all the 105 plant MIPs from the three plant species in the loop B region. The first six sequences correspond to the experimentally determined aquaporin structures from different species. Their respective PDB IDs are shown in the first column. The beginning and end residue numbers are also given for each PDB structure. Small and weakly polar residues (Gly, Ala, Thr, Ser and Cys) occurring in the helix-helix interfaces are shaded in gray color. Click here for file

Additional File 8 **Structure-based sequence alignment of plant MIPs in the loop E region**. Structure-based sequence alignments are provided for all the 105 plant MIPs from the three plant species in the loop E region. The first six sequences correspond to the experimentally determined aquaporin structures from different species. Their respective PDB IDs are shown in the first column. The beginning and end residue numbers are also given for each PDB structure. Small and weakly polar residues (Gly, Ala, Thr, Ser and Cys) occurring in the helix-helix interfaces are shaded in gray color. The residues forming arginine/aromatic selectivity filter are shown in bold. Click here for file

## Figures and Tables

**Table 1:** Six additional rice MIP sequences. Details of new rice MIP genes identified from the TBLASTN search.

Name	Accession no.^a^	Length	Comments
OsPIP1;4	AAB82140	289	Belongs to *indica *cultivar-subspecies
OsPIP1;5	AAB18817	291	Belongs to *indica *cultivar-subspecies
OsTIP2;3	CAD41593	768	Has ~500 residue long C-terminal extension
OsNIP1;5	9629.t00960	303	TIGR Release 4 has 70 residue deletion between the two NPA regions
OsNIP3;4	9640.t00931	267	N-terminus is longer by 31 residues in TIGR release 4
OsNIP3;5	BAC98553	283	

^a ^Accession codes of NIP1;5 and NIP3;4 are from TIGR; all others are GenBank accession codes

**Table 2:** Residues in the helix-helix interfaces of aquaporin crystal structures. Residue pairs that occur at the helix-helix interface in the six high-resolution aquaporin crystal structures.

Helix Pairs	Residues from the crystal structures^a^	<dist.>^b^(Å)
TM1...TM2	**S28**...F58, **G25**...W48, **G19**...F43, **T55**...F81,**G27**...F48, **G22**...F62	4.86

TM1...TM3	M21...Q103, **T18**...Q93, **T12**...Q88, **T48**...Q126, **T20**...Q93, **T15**...Q107	5.74
	F18...**G106**, F15...**G96**, C9...**G91**, F45...**G129**, F17...**G9**6, F12...**G110**	4.55
	I25...**A107**, I22...**A97**, V16...**G92 **L52...**A130**, V24...**A97**, V19...**A111**	5.70
	I25...**A110**, I22...**A100**, V16...**A95**, L52...**G133**, V24...**G100**, V19...**G114**	5.52

TM4...TM6	**T148**...P218, **T156**...P240, **S142**...P212, **T172**...P249, **T138**...P208, **T154**...P229	5.29
	**A157**...Y229, **A165**...Y251, **G151**...Y223, **S181**...H260, **A147**...Y219, **G163**...Y240	7.73
	L141/I145...**G217**, F149/M153...**G239**, L135/L139...V211, L165/I169...**G248**, T131/I135...**G207**, M147/V151...**G228**	5.31
	I145/L149...**G221**, M153/A157...**G243**, L139/A143...**G215**, I169/F173...**G252**, I135/L139...**G211**, V151/F155...**G232**	4.35
	L149/V152...**A225**, A157/M160...**G247**, A143/L146...**G219**, F173/V176...**A256**, L139/V142...**G215**, F155/M158...**A236**	5.17
	V152...**A222**, M160...**A244**, L146...**G216**, V176...**A253**, V142...**A212**, M158...**A233**	5.51

TM2...TM5	**S55**...L183, **S45**...A192, **A40**...L175, **A78**...L211, **A45**...L173, **G59**...T188	5.91
	**G59...G175**,**G59...G184**,**G44...G167**, **G82...G203**,**G49...G165**,**G63...G180**	4.31

LB...TM4	**G84**...V152, **A74**...M160, **G69**...L146, **G107**...V176, **A74**...V142, **G88**...M158	6.96

LB...TM6	**A80...A222**,**A70...A244**,**A65...G216**, **A103...A253**,**A70...A212**,**A84...A233**	5.75

LE...TM3	**G200...A107**,**G209...A97**,**A192...G92**, **G228...A130**,**A190...A97**,**G205...A111**	4.89

LE...TM6	**S198**...F214, D207...P236, **S190**...F208, **S226**...F245,**S188**...Y204, **T203**...Y225	6.52

^a ^Residue pairs are given respectively for the crystal structures 1J4N, 1FX8, 1RC2, 1Z98, 2B6O and 2F2B. For each residue given for 1J4N, the equivalent residues from the other crystal structures were obtained from structure-based sequence alignment (see Additional files 1 to 8). For example, the equivalent residue pairs for (S28, F58) in 1J4N are (G25, W48) in 1FX8, (G19, F43) in 1RC2, (T55, F81) in 1Z98, (G27, F48) in 2B6O and (G22, F62) in 2F2B. Small residues (Gly/Ala/Ser/Thr) are shown in bold. ^b ^Average Cα...Cα distances calculated for residue pairs from all the six crystal structures are given.

**Table 3:** Conservation of small and weakly polar residues at the helix-helix interfaces of plant MIP proteins

Residue^a^	All families ^b, c^	PIPs^c^	TIPs^c^	NIPs^c^	SIPs^c^
T48	T(93), A, S **(*97%*)**	T(100) **(*100%*)**	T(94) **(*94%*)**	T(92), S **(*96%*)**	T(63), A(25), S **(*100%*)**
T55	G(39), T(35), A **(*93%*)**	T(85), S **(*100%*)**	G(78), A **(*100%*)**	G(50), T, A **(*81%*)**	G(38), S(25), A **(*75%*)**
A78	A(77), S, C, T **(*93%*)**	A(95), S **(*98%*)**	A(88), S **(*100%*)**	A(62), C, S **(*96%*)**	T(38) **(*38%*)**
G82	G(63), A(28), S, T **(*94%*)**	G(100) **(*100%*)**	A(91), G, T **(*100%*)**	G(96) **(*96%*)**	S(38) **(*38%*)**
A103	A(88), S, T, C **(*96%*)**	A(100) **(*100%*)**	A(97) **(*97%*)**	A(85), S(15) **(*100%*)**	T(50), C **(*62%*)**
G107	G(61), A(32), S, T **(*100%*)**	G(97), A **(*100%*)**	G(78), A, S **(*100%*)**	A(77), T, S, G **(*100%*)**	A(100) **(*100%*)**
G129	G(90), A **(*100%*)**	G(100) **(*100%***)	G(81), A **(*100%*)**	G(81), A **(*100%*)**	G(100) **(*100%*)**
A130	A(63), S(36), G **(*100%*)**	A(97), G **(*100%*)**	S(59), A(41) **(*100%*)**	S(65), A(35) **(*100%*)**	A(75), S(25) **(*100%*)**
G133	A(55), G(44) **(*99%*)**	G(97) **(*97%*)**	A(97), G **(*100%*)**	A(100) **(*100%*)**	G(88), A **(*100%*)**
T172	T(91), S, A **(*100%*)**	T(100) **(*100%*)**	T(97), A **(*100%*)**	T(73), S(27) **(*100%*)**	T(75), S(25) **(*100%*)**
S181	A(40), S(40), G, T **(*93%*)**	S(100) **(*100%*)**	A(84), S, T **(*94%*)**	A(58), G(35), S, T **(*100%*)**	T(25), G **(*38%*)**
G203	G(91), A, S **(*99%*)**	G(100) **(*100%*)**	G(100) **(*100%*)**	G(92), A **(*96%*)**	S(75), A(25) **(*100%*)**
S226	S(71), T, A **(*97%*)**	S(97), T **(*100%*)**	S(75), A **(*94%*)**	T(54), S(46) **(*100%*)**	A(88) **(*88%*)**
G248	G(78), A, S, C **(*100%*)**	G(100) **(*100%*)**	G(100) **(*100%*)**	A(50), G(38), S **(*100%*)**	C(38), A(25), S(25), G **(*100%*)**
G252	G(97), A **(*100%*)**	G(100) **(*100%*)**	G(100) **(*100%*)**	G(100) **(*100%*)**	G(62), A(38) **(*100%*)**
A253	A(66), G, T, S **(*97%*)**	A(97), G **(*100%*)**	G(72), A(25), S **(*100%*)**	A(69), T **(*88%*)**	A(62), T(38) **(*100%*)**
A256	A(71), G(27) **(*98%*)**	A(97) **(*97%*)**	A(100) **(*100%*)**	G(100) **(*100%*)**	A(62), G(25), S **(*100%*)**

^a ^Residue numbers correspond to that of the plant aquaporin SoPIP2;1 (PDB ID: 1Z98).

^b ^Conservation is reported for all MIP subfamilies from the structure-based sequence alignments of 105 MIP sequences from rice, maize and *Arabidopsis*.

^c ^Small and weakly polar interfacial residues in plant MIP sequences and their conservation (if it exceeds 25%) are given. If the conservation is less than 25%, only the residues are reported. Group conservation of small and weakly polar residues (Gly, Ala, Ser, Thr and Cys) is shown in bold and italics.

**Table 4:** Aromatic/Arginine signatures that are identical or similar in *Arabidopsis*, rice and maize

MIP Members	H2	H5	LE1	LE2
**PIP family**				
All OsPIPs All ZmPIPs All AtPIPs	F	H	T	R

**TIP family**				
OsTIP1;1, OsTIP1;2 ZmTIP1;1, ZmTIP1;2 AtTIP1;1, AtTIP1;2, AtTIP1;3	H	I	A	V
OsTIP2;1, OsTIP2;2, OsTIP2;3, OsTIP3;1, OsTIP3;2, OsTIP4;3 ZmTIP2;1, ZmTIP2;2, ZmTIP2;3, ZmTIP3;1, ZmTIP4;4 AtTIP2;1, AtTIP2;2, AtTIP2;3, AtTIP3;1, AtTIP3;2, AtTIP4;1	H	I/M/V	G/A/S	R
AtTIP5;1^a^	N	V	G	C

**NIP family**				
OsNIP1;1, OsNIP1;2, OsNIP1;3, OsNIP1;4, OsNIP1;5 ZmNIP1;1 AtNIP1;1, AtNIP1;2, AtNIP2;1, AtNIP4;1, AtNIP4;2	W	V	A	R
OsNIP3;1, OsNIP3;3 ZmNIP3;1 AtNIP5;1, AtNIP6;1, AtNIP7;1	A	I/V	A/G	R

**SIP family**				
AtSIP1;1, AtSIP1;2^a^	I/V	F/V	P	I
	(T	F/V/I	P	I)^b^
OsSIP2;1 ZmSIP2;1 AtSIP2;1	S	H	G	A/S

^a ^These selectivity filters are found only in *Arabidopsis*

^b^ar/R selectivity filter reported by Wallace and Roberts [24].

**Table 5:** Ar/R signatures that are unique to rice and maize and are not found in *Arabidopsis*

MIP Members	H2	H5	LE1	LE2
**TIP family**				
ZmTIP4;1, ZmTIP4;2	H	S	A	R
OsTIP4;2, ZmTIP4;3	Q	S/T	A	R
OsTIP5;1, ZmTIP5;1	Q	V	A	R
OsTIP4;1	T	T	A	R

**NIP family**				
OsNIP2;1, OsNIP2;2, ZmNIP2;1, ZmNIP2;2	G	S	G	R
OsNIP3;2	A	A	A	R
OsNIP4;1	C	G	G	R
OsNIP3;5	A	A	P	R

**SIP family**				
OsSIP1;1, ZmSIP1;1, ZmSIP1;2	L	I/V	P	N

**Table 6:** Percentage sequence identities between plant MIP subfamilies and the three template sequences

Template^a^	PIPs	TIPs	NIPs	SIPs
	**Rice**			

AQP1	38.2–44.8	31.4–40.2	25.9–34.7	23.4–25.7
GlpF	31.0–35.4	29.2–36.3	25.5–38.5	22.0–29.4
AqpZ	27.2–35.5	30.1–38.3	28.6–39.9	26.7–29.1

	**Maize**			

AQP1	40.1–46.0	30.0–37.9	29.0–32.2	25.2–26.1
GlpF	31.6–34.5	29.7–33.8	32.3–35.5	22.9–26.5
AqpZ	27.8–36.2	30.8–38.1	33.2–40.4	24.3–26.0

	***Arabidopsis***			

AQP1	40.9–45.3	29.6–40.4	26.6–33.2	24.2–26.8
GlpF	29.2–35.3	26.9–33.2	32.3–39.3	24.8–29.6
AqpZ	29.4–34.4	27.2–38.5	32.6–39.2	22.4–24.0

^a ^Amino acid sequences corresponding to AQP1 (bovine), GlpF (*E. coli*) and AqpZ (*E. coli*) were used for this analysis. The corresponding PDB codes of these proteins are 1J4N, 1FX8 and 1RC2 and they served as template structures for the plant MIP modeling.
