# Supplementary material for: Homology modeling of major intrinsic proteins in rice, maize and Arabidopsis: comparative analysis of transmembrane helix association and aromatic/arginine selectivity filters
Source: BMC Struct Biol. 2007 Apr 19;7:27. doi: 10.1186/1472-6807-7-27 (PMC1866351; doi:10.1186/1472-6807-7-27)
Supplement: Additional File 1 — Structure-based sequence alignment of plant MIPs in TM1 region. Structure-based sequence alignments are provided for all the 105 plant MIPs from the three plant species in the TM1 region. The first six sequences correspond to the experimentally determined aquaporin structures from different species. Their respective PDB IDs are shown in the first column. The beginning and end residue numbers are also given for each PDB structure. Small and weakly polar residues (Gly, Ala, Thr, Ser and Cys) occurring in the helix-helix interfaces are shaded in gray color. [file 1472-6807-7-27-S1.pdf]

Structure-based sequence alignment of TM1

```
Helix 1  PIP

1J4N                  10 FWRAVVAEFLAMILFIFISIGSALGF 35
1FX8                   7 LKGQCIAEFLGTGLLIFFGVGCVAAL 32
1RC2                   1 MFRKLAAECFGTFWLVFGGCGSAVLA 26
1Z98                  37 FWRAAIAEFIATLLFLYITVATVIGH 62
2B6O                   9 FWRAIFAEFFATLFYVFFGLGASLRW 34
2F2B                   4 LTKRCIAEFIGTFILVFFGAGSAAVT 29
OsPIP1;1                 FYRAGIAEFVATFLFLYITILTVMGV
OsPIP1;2                 FYRAGIAEFMATFLFLYITVLTVMGV
OsPIP1;3                 FYRAGIAEFVATFLFLYISILTVMGV
OsPIP1;4                 FYRAGIADFVATFLFLYITILTVMGV
OsPIP1;5                 FYRAGIAEFMATFLFLYITVLTVMGV
OsPIP2;1                 LYRAVIAEFIATLLFLYITVATVIGY
OsPIP2;2                 LYRAVIAEFIATLLFLYITVATVIGY
OsPIP2;3                 LYRAVIAEFVATLLFLYITVATVIGY
OsPIP2;4                 LYRALIAEFVATLLFLYVTVATVIGY
OsPIP2;5                 LYRAVIAEFVATLLFLYVTVATVIGY
OsPIP2;6                 FYRALIAEFIATLLFLYITVATVIGY
OsPIP2;7                 LYRALIAEFMATLIFLYVSIATVIGY
OsPIP2;8                 LYRAAIAEFTATLLLVCISVSTVIGE
ZmPIP1;1                 FYRPGIAEFVATFLFLYISILTVMGV
ZmPIP1;2                 FYRAGIAEFVATFLFLYITILTVMGV
ZmPIP1;3                 FYRAGIAEFVATFLFLYITVLTVMGV
ZmPIP1;4                 FYRAGIAEFVATFLFLYITVLTVMGV
ZmPIP1;5                 FYRAGIAEFVATFLFLYISILTVMGV
ZmPIP1;6                 FYRAGIAEFVATFLFLYVTVLTVMGV
ZmPIP2;1                 LYRAVIAEFIATLLFLYITVATVIGY
ZmPIP2;2                 LYRAVIAEFIATLLFLYVTVATVIGY
ZmPIP2;3                 LYRAVIAEFIATLLFLYITVATVIGY
ZmPIP2;4                 LYRAVIAEFIATLLFLYITVATVIGY
ZmPIP2;5                 LYRAVIAEFVATLLFLYITVATVIGY
ZmPIP2;6                 LYRAVIAEFVATLLFLYITVATVIGY
ZmPIP2;7                 LYRAAIAEFIATLLFLYITVLTIIGY
AtPIP1;1                 FWRAGIAEFIATFLFLYITVLTVMGV
AtPIP1;2                 FWRAGIAEFIATFLFLYITVLTVMGV
AtPIP1;3                 FYRAGIAEFIATFLFLYITVLTVMGV
AtPIP1;4                 FYRAGIAEFIATFLFLYITVLTVMGV
AtPIP1;5                 FYRAGIAEFIATFLFLYVTVLTVMGV
AtPIP2;1                 FYRAVIAEFVATLLFLYITVLTVIGY
AtPIP2;2                 LYRAVIAEFVATLLFLYITVLTVIGY
AtPIP2;3                 LYRAVIAEFVATLLFLYVTVLTVIGY
AtPIP2;4                 LYRAVIAEFVATLLFLYVSILTVIGY
AtPIP2;5                 FYRALIAEFIATLLFLYVTIMTVIGY
AtPIP2;6                 FYRAVIAEFIATLLFLYVTVLTVIGF
AtPIP2;7                 FYRALIAEFIATLLFLYVTVATVIGH
AtPIP2;8                 FYRAIIAEFIATLLFLYVTVATVIGH
```

Helix1   TIP

```
1J4N        10 FWRAVVAEFLAMILFIFISIGSALGF 35
1FX8         7 LKGQCIAEFLGTGLLIFFGVGCVAAL 32
1RC2         1 MFRKLAAECFGTFWLVFGGCGSAVLA 26
1Z98        37 FWRAAIAEFIATLLFLYITVATVIGH 62
2B6O         9 FWRAIFAEFFATLFYVFFGLGASLRW 34
2F2B         4 LTKRCIAEFIGTFILVFFGAGSAAVT 29
OsTIP1;1       ALKAALAEFISTLIFVFAGQGSGMAF
OsTIP1;2       TAKAAVAEFISMLIFVFAGSGSGMAF
OsTIP2;1       SVKAYVAEFIATLLFVFAGVGSAIAY
OsTIP2;2       SLKAYVAEFISTLVFVFAGVGSAIAY
OsTIP2;3       SIKAYVAEFIATLLFVFAGVGSAIAY
OsTIP3;1       TIRAAISEFLATAIFVFAAEGSILSL
OsTIP3;2       ATRAALSEFVATAVFVFAAEGSVYGL
OsTIP4;1       CVRAVLAELVLTFVFVFTGVAATMAA
OsTIP4;2       CLRAVAGELLFTFLFVFIGVASTITA
OsTIP4;3       CLRAVVAELLLTFLFVFSGVGSAMAA
OsTIP5;1       ALRAYFAEFFSTFLFVFIAVGSTISA
ZmTIP1;1       ALKAAFAEFISTLIFVFAGQGSGMAF
ZmTIP1;2       TAKAAVAEFISTLIFVFAGSGSGMAF
ZmTIP2;1       SIKAYVAEFIATLLFVFAGVGSAIAY
ZmTIP2;2       SIKAYVAEFIATLLFVFAGVGSAIAF
ZmTIP2;3       SLKAYVAEFIATLLFVFAGVGSAIAY
ZmTIP3;1       TIRAAISEFIATAIFVFAAEGSVLSL
ZmTIP4;1       CVRAVLAELVLTFLFVFTGVSAAMAA
ZmTIP4;2       CVRAVLAELVLTFLFVFTGVSASMAA
ZmTIP4;3       FFRGVLGELVLTFLFVFIGVGAAMTD
ZmTIP4;4       CVRAVLAELILTFLFVFAGVGSAMAT
ZmTIP5;1       SLRSYLAEFISTFLFVFTAVGSAISA
AtTIP1;1       ALKAALAEFISTLIFVVAGSGSGMAF
AtTIP1;2       ALRAALAEFISTLIFVFAGSGSGIAF
AtTIP1;3       AIRAAFAEFFSMVIFVFAGQGSGMAY
AtTIP2;1       SLRAYLAEFISTLLFVFAGVGSAIAY
AtTIP2;2       SLKAYLSEFIATLLFVFAGVGSALAF
AtTIP2;3       SLKAYLSEFIATLLFVFAGVGSAVAF
AtTIP3;1       SIRATLAEFLSTFVFVFAAEGSILSL
AtTIP3;2       SIRATLAEFLSTFVFVFAGEGSILAL
AtTIP4;1       CIKALIVEFITTFLFVFAGVGSAMAT
AtTIP5;1       ALRCYVSEFISTFFFVLAAVGSVMSS
```

Helix1 NIP

1J4N	10	FWRAVVAEFLAMILFIFISIGSALGF	35
1FX8	7	LKGQCIAEFLGTGLLIFFGVGCVAAL	32
1RC2	1	MFRKLAAECFGTFWLVFGGCGSAVLA	26
1Z98	37	FWRAAIAEFIATLLFLYITVATVIGH	62
2B6O	9	FWRAIFAEFFATLFYVFFGLGASLRW	34
2F2B	4	LTKRCIAEFIGTFILVFFGAGSAAVT	29
OsNIP1;1		FIQKIIAEIFGTYFLIFAGCGAVTIN	
OsNIP1;2		KNLLILAEILGTYFMIFAGCGAVVVN	
OsNIP1;3		FAQKVIAEILGTFFLIFAGCAAVAVN	
OsNIP1;4		FLQMLLAEFLATFFLMFAGLGAITVE	
OsNIP1;5		FMQKILTEILGTYFMIFAGCGAVVVN	
OsNIP2;1		LLKKVVSEVVATFLLVFMTCGAAGIS	
OsNIP2;2		LLKKVISEVVATFLLVFVTCGAASIY	
OsNIP3;1		LHVCLGAEFVGTFILIFFATAAPIVN	
OsNIP3;2		LVKKVGAEFFGTFTLIFTVLSTIIMD	
OsNIP3;3		LLKKVSAEFFGTFILIFTVLSTIIMD	
OsNIP3;4		LAKKAAAEFVGTFILIFAMLSTIVTD	
OsNIP3;5		LVKKAAAEFIGTFILVFTVLSTVVMD	
OsNIP4;1		LIREVMVEGLASFLVVFWSCVAALMQ	
ZmNIP1;1		FIQKIIAEIFGTYFLMFAGCGAVTIN	
ZmNIP2;1		LLKKVVSEVVSTFLLVFVTCGAAGIY	
ZmNIP2;2		LGKKVISEVVATFLLVFVTCGAASIY	
ZmNIP3;1		LTRKLGAEFVGTFILIFFATAAPIVN	
AtNIP1;1		FLQKLIAEFLGTYFLVFTGCASVVVN	
AtNIP1;2		FLQKLMAEVLGTYFLIFAGCAAVAVN	
AtNIP2;1		FLQKLLAELVGTYYLIFAGCAAIAVN	
AtNIP3;1		FVQKLIGEFVGTFTMIFAGCSAIVVN	
AtNIP4;1		LTQKLIAEMIGTYFIVFSGCGVVVVN	
AtNIP4;2		LTQKLIAEMIGTYFIIFSGCGVVVVN	
AtNIP5;1		LTRKLGAEFVGTFILIFTATAGPIVN	
AtNIP6;1		LYRKLGAEFVGTLILIFAGTATAIVN	
AtNIP7;1		PLRIVMAELVGTFILMFSVCGVISST	

Helix1 SIP

		10 35	
1J4N	10	FWRAVVAEFLAMILFIFISIGSALGF	35
1FX8	7	LKGQCIAEFLGTGLLIFFGVGCVAAL	32
1RC2	1	MFRKLAAECFGTFWLVFGGCGSAVLA	26
1Z98	37	FWRAAIAEFIATLLFLYITVATVIGH	62
2B6O	9	FWRAIFAEFFATLFYVFFGLGASLRW	34
2F2B	4	LTKRCIAEFIGTFILVFFGAGSAAVT	29
OsSIP1;1		AVRAAAADAAVTFLWVLCVSTLGAST	
OsSIP2;1		RPWLVVGDLVVAAMWVCAGALVKLAV	
ZmSIP1;1		TVRAAAADAVVTFLWVLCASALGAST	
ZmSIP1;2		ALRAAAADAVVTFLWVLCVSTLGAST	
ZmSIP2;1		RPWLVVGDLALAAAWVCAGALVKLLV	
AtSIP1;1		VLKSAIGDMLMTFSWVVLSATFGIQT	
AtSIP1;2		AVKSALGDMVITFLWVILSATFGIQT	
AtSIP2;1		RIGLVVTDLVLSFMWIWAGVLVNILV	
