# Supplementary material for: Homology modeling of major intrinsic proteins in rice, maize and Arabidopsis: comparative analysis of transmembrane helix association and aromatic/arginine selectivity filters
Source: BMC Struct Biol. 2007 Apr 19;7:27. doi: 10.1186/1472-6807-7-27 (PMC1866351; doi:10.1186/1472-6807-7-27)
Supplement: Additional File 2 — Structure-based sequence alignment of plant MIPs in TM2 region. Structure-based sequence alignments are provided for all the 105 plant MIPs from the three plant species in the TM2 region. The first six sequences correspond to the experimentally determined aquaporin structures from different species. Their respective PDB IDs are shown in the first column. The beginning and end residue numbers are also given for each PDB structure. Small and weakly polar residues (Gly, Ala, Thr, Ser and Cys) occurring in the helix-helix interfaces are shaded in gray color. The residues forming arginine/aromatic selectivity filter are shown in bold. [file 1472-6807-7-27-S2.pdf]

<u>Structure-based sequence alignment of TM2</u>

Helix 2 PIP

1J4N	50	DNVKVSLA**F**GLSIATLAQSVGH	71
1FX8	40	GQWEISVI**W**GLGVAMAIYLTAG	61
1RC2	35	GFAGVALA**F**GLTVLTMAFAVGH	56
1Z98	73	GLLGIAWA**F**GGMIFVLVYCTAG	94
2B6O	40	HVLQVALA**F**GLALATLVQAVGH	61
2F2B	54	DWVAIGLA**F**GFAIAASIYALGN	75
OsPIP1;1		GIQGIAWS**F**GGMIFALVYCTAG	
OsPIP1;2		GIQGIAWS**F**GGMIFALVYCTAG	
OsPIP1;3		GIQGIAWS**F**GGMIFALVYCTAG	
OsPIP1;4		GIQGIPWS**F**GSMIFALVYCTAG	
OsPIP1;5		GIQGIAWS**F**GGMIFALVYCTAG	
OsPIP2;1		GVLGIAWA**F**GGMIFILVYCTAG	
OsPIP2;2		GILGIAWA**F**GGMIFILVYCTAG	
OsPIP2;3		GILGIAWA**F**GGMIFILVYCTAG	
OsPIP2;4		GVLGIAWA**F**GGMIFILVYCTAG	
OsPIP2;5		GVLGIAWA**F**GGMIFILVYCTAG	
OsPIP2;6		GTLGIAWA**F**GGMIFILVYCTAG	
OsPIP2;7		GYLGVAWS**F**GATIFVLVYCTGG	
OsPIP2;8		GVLGIAWA**F**GGLIFVLVYCTAG	
ZmPIP1;1		GIQGIAWS**F**GGMILALVYCTAG	
ZmPIP1;2		GIQGIAWS**F**GGMIFALVYCTAG	
ZmPIP1;3		GIQGIAWS**F**GGMIFALVYCTAG	
ZmPIP1;4		GIQGIAWS**F**GGMIFALVYCTAG	
ZmPIP1;5		GIQGIAWS**F**GGMIFALVYCTAG	
ZmPIP1;6		GIQGIAWA**F**GGMIFALVYCTAG	
ZmPIP2;1		GVLGIAWA**F**GGMIFVLVYCTAG	
ZmPIP2;2		GVLGIAWA**F**GGMIFVLVYCTAG	
ZmPIP2;3		GILGIAWA**F**GGMIFILVYCTAG	
ZmPIP2;4		GILGIAWA**F**GGMIFILVYCTAG	
ZmPIP2;5		GVLGIAWA**F**GGMIFILVYCTAG	
ZmPIP2;6		GILGIAWA**F**GGMIFILVYCTAG	
ZmPIP2;7		GILGIAWA**F**GGMIFILVYCTAG	
AtPIP1;1		GIQGIAWA**F**GGMIFALVYCTAG	
AtPIP1;2		GIQGIAWA**F**GGMIFALVYCTAG	
AtPIP1;3		GIQGIAWA**F**GGMIFALVYCTAG	
AtPIP1;4		GIQGIAWA**F**GGMIFALVYCTAG	
AtPIP1;5		GIQGIAWA**F**GGMIFALVYCTAG	
AtPIP2;1		GILGIAWA**F**GGMIFILVYCTAG	
AtPIP2;2		GILGIAWA**F**GGMIFILVYCTAG	
AtPIP2;3		GILGIAWA**F**GGMIFILVYCTAG	
AtPIP2;4		GILGIAWA**F**GGMIFVLVYCTAG	
AtPIP2;5		GVLGIAWA**F**GGMIFILVYCTAG	
AtPIP2;6		GLLGISWA**F**GGMIFILVYCTAG	
AtPIP2;7		GLLGIAWA**F**GGMIFVLVYCTAG	
AtPIP2;8		GLLGIAWA**F**GGMIFVLVYCTAG	

Helix 2 TIP

1J4N	50	DNVKVSLA**F**GLSIATLAQSVGH	71
1FX8	40	GQWEISVI**W**GLGVAMAIYLTAG	61
1RC2	35	GFAGVALA**F**GLTVLTMAFAVGH	56
1Z98	73	GLLGIAWA**F**GGMIFVLVYCTAG	94
2B6O	40	HVLQVALA**F**GLALATLVQAVGH	61
2F2B	54	DWVAIGLA**F**GFAIAASIYALGN	75
OsTIP1;1		GLIAAAVA**H**AFALFVAVSVGAN	
OsTIP1;2		GLIAASLA**H**ALALFVAVAVGAN	
OsTIP2;1		GLVAIAIA**H**ALALFVGVSVAAN	
OsTIP2;2		GLVAVAVC**H**GFGLFVAVAIGAN	
OsTIP2;3		GLVAIAIA**H**AFALFVGVSMAAN	
OsTIP3;1		GLVAVSLA**H**ALALAVAVAVAVN	
OsTIP3;2		GLLVVAVA**H**ALALAAAVAVSRN	
OsTIP4;1		ALAGVAIA**T**ALAAGVLVTAGFH	
OsTIP4;2		AVTAAAMA**Q**ALVVAVLATAGFH	
OsTIP4;3		GLTAVAAA**H**ALVVAVMVSAGLH	
OsTIP5;1		SLMATAVA**Q**AFGLFAAVFIAAD	
ZmTIP1;1		GLIAAAVA**H**AFALFVAVSVGAN	
ZmTIP1;2		GLIAASLA**H**ALALFVAVSVGAN	
ZmTIP2;1		GLVAIAIA**H**ALALFVGVSVAAN	
ZmTIP2;2		GLVAIAVA**H**ALALFVGVSVAAN	
ZmTIP2;3		GLVAIAIA**H**AFALFVGVSMAAN	
ZmTIP3;1		GLVAVALA**H**ALALAVAVAVAVN	
ZmTIP4;1		TLAAVAIA**H**ALAAGVLVTAGFH	
ZmTIP4;2		TLAAVAIA**H**ALAAGVLVTAGFH	
ZmTIP4;3		DLTAVALG**Q**ALVVAVIATAGFH	
ZmTIP4;4		GLTAVALA**H**TLVVAVMVSAGLH	
ZmTIP5;1		PLVATAVA**Q**AFGLFAAVLIAAD	
AtTIP1;1		GLVAAAVA**H**AFGLFVAVSVGAN	
AtTIP1;2		GLVAAALA**H**AFGLFVAVSVGAN	
AtTIP1;3		GLVAASLS**H**AFALFVAVSVGAN	
AtTIP2;1		GLVAIAVC**H**GFALFVAVAIGAN	
AtTIP2;2		GLVAVAVA**H**AFALFVGVSIAAN	
AtTIP2;3		GLVAIAIA**H**AFALFVGVSIAAN	
AtTIP3;1		GLILVALA**H**AFALFAAVSAAIN	
AtTIP3;2		GLVLVALA**H**ALALFAAVSAAIN	
AtTIP4;1		GLFAVAVA**H**AFVVAVMISAG-H	
AtTIP5;1		GVLIPAIA**N**ALALSSSVYISWN	

Helix 2 NIP

1J4N	50	DNVKVSLA**F**GLSIATLAQSVGH	71
1FX8	40	GQWEISVI**W**GLGVAMAIYLTAG	61
1RC2	35	GFAGVALA**F**GLTVLTMAFAVGH	56
1Z98	73	GLLGIAWA**F**GGMIFVLVYCTAG	94
2B6O	40	HVLQVALA**F**GLALATLVQAVGH	61
2F2B	54	DWVAIGLA**F**GFAIAASIYALGN	75
OsNIP1;1		TFPGVAIV**W**GLAVMVMVYAVGH	
OsNIP1;2		TFPGICAV**W**GLVVMVLVYTVSH	
OsNIP1;3		TFPGICIT**W**GLAVMVMVYSVGH	
OsNIP1;4		TFPGVAVA**W**GAAVMAMVYAVGH	
OsNIP1;5		MFPGICVV**W**GLVVTVLVYSVGH	
OsNIP2;1		SQLGQSIA**G**GLIVTVMIYAVGH	
OsNIP2;2		SQLGQSVV**G**GLIVTVMIYATGH	
OsNIP3;1		SPFGNAAC**A**GLAVTTIILSTGH	
OsNIP3;2		SLLGIATS**A**GLAVTVLVLSLIH	
OsNIP3;3		TLLGIATS**A**GLAVTVLVLSLIH	
OsNIP3;4		GLVGVAAS**I**GLAVAVLVMSLAH	
OsNIP3;5		TLVGVAAS**A**GLAVVAVVLSVVH	
OsNIP4;1		GTLTFPMV**C**LVVAMTVAFVLSW	
ZmNIP1;1		TFPGVAIV**W**GLAVMVMVYAVGH	
ZmNIP2;1		SQLGQSVA**G**GLIVTVMIYAVGH	
ZmNIP2;2		SQLGQSVA**G**GLIVTVMIYATGH	
ZmNIP3;1		SPFGNAAC**A**GLAVATVILSTGH	
AtNIP1;1		TLPGIAIV**W**GLTIMVLIYSLGH	
AtNIP1;2		TLPGIAIV**W**GLTVMVLVYSLGH	
AtNIP2;1		TLVGIAVV**W**GIVIMVLVYCLGH	
AtNIP3;1		TLPGIALV**W**GLVVTVMIYSIGH	
AtNIP4;1		TFPGICVT**W**GLIVMVMIYSTGH	
AtNIP4;2		TFPGICVT**W**GLIVMVMIYSTGH	
AtNIP5;1		TLIGNAAC**A**GLAVMIIILSTGH	
AtNIP6;1		TLIGCAAS**A**GLAVMIVILSTGH	
AtNIP7;1		GLLEYAVT**A**GLSVVVVVYSIGH	

Helix 2 SIP

1J4N	50	DNVKVSLA**F**GLSIATLAQSVGH	71
1FX8	40	GQWEISVI**W**GLGVAMAIYLTAG	61
1RC2	35	GFAGVALA**F**GLTVLTMAFAVGH	56
1Z98	73	GLLGIAWA**F**GGMIFVLVYCTAG	94
2B6O	40	HVLQVALA**F**GLALATLVQAVGH	61
2F2B	54	DWVAIGLA**F**GFAIAASIYALGN	75
OsSIP1;1		ALLVTVSL**L**SVLLFAFNLLCDA	
OsSIP2;1		ADAVKVAL**S**LVYMFFFAWLEGF	
ZmSIP1;1		ALLVTTSL**L**SVLLFTFDLLCGA	
ZmSIP1;2		ALLVTVSL**L**SVLLFVFNILCDA	
ZmSIP2;1		AEAVKVSL**S**LVYMFLFAWLEAA	
AtSIP1;1		PLVILTSL**I**FVYVSIFTVIF--	
AtSIP1;2		PLVISTLV**V**FVSISIFTVIGNV	
AtSIP2;1		GEIVRYLF**S**IISMFIFAYLQQA	
