# Supplementary material for: Homology modeling of major intrinsic proteins in rice, maize and Arabidopsis: comparative analysis of transmembrane helix association and aromatic/arginine selectivity filters
Source: BMC Struct Biol. 2007 Apr 19;7:27. doi: 10.1186/1472-6807-7-27 (PMC1866351; doi:10.1186/1472-6807-7-27)
Supplement: Additional File 3 — Structure-based sequence alignment of plant MIPs in TM3 region. Structure-based sequence alignments are provided for all the 105 plant MIPs from the three plant species in the TM3 region. The first six sequences correspond to the experimentally determined aquaporin structures from different species. Their respective PDB IDs are shown in the first column. The beginning and end residue numbers are also given for each PDB structure. Small and weakly polar residues (Gly, Ala, Thr, Ser and Cys) occurring in the helix-helix interfaces are shaded in gray color. [file 1472-6807-7-27-S3.pdf]

Structure-based sequence alignment of TM3

```
Helix 3  PIP

1J4N                    92 SVLRAIMYIIAQCVGAIVATAILSG 116
1FX8                    82 DKRKVIPFIVSQVAGAFCAAALVYG 106
1RC2                    77 PAKEVVGYVIAQVVGGIVAAALLYL 101
1Z98                   115 SLLRALVYMIAQCLGAICGVGLVKA 139
2B6O                    82 SLLRAICYVVAQLLGAVAGAAVLYS 106
2F2B                    96 PGREVVPYIIAQLLGAAFGSFIFLQ 120
OsPIP1;1                   SLTRAIFYIVMQCLGAICGAGVVKG
OsPIP1;2                   SLTRALFYMVMQCLGAICGAGVVKG
OsPIP1;3                   SLTRAVFYMAMQCLGAICGAGVVKG
OsPIP1;4                   SLSRAIFYILMQCLGAICRTGFVKG
OsPIP1;5                   SLTRALFYMVMQCLGAICGAGVVKG
OsPIP2;1                   SLVRAILYIVAQCLGAICGVGLVKA
OsPIP2;2                   SLIRAVLYIIAQCLGAICGVGLVKG
OsPIP2;3                   SLVRAVLYIVAQSLGAICGVGLVKG
OsPIP2;4                   SLVRALLYMAAQCLGAICGVALVKG
OsPIP2;5                   SLVRAILYIVAQCLGAVCGVALVKG
OsPIP2;6                   SVIRAVMYIVAQCLGGIVGVGIVKG
OsPIP2;7                   SLVRTVLYVVAQCLGAIAGAGIVKG
OsPIP2;8                   SLPRAALYTMAQCVGAVCGAGLARA
ZmPIP1;1                   SLTRAVFYIIMQCLGAICGRGVVKG
ZmPIP1;2                   SLTRALFYIIMQCLGAVCGAGVVKG
ZmPIP1;3                   SLTRAIFYIIMQCLGAICGAGVVKG
ZmPIP1;4                   SLTRAIFYIIMQCLGAICGAGVVKG
ZmPIP1;5                   SLTRALFYMVMQCLGAICGAGVVKG
ZmPIP1;6                   SLTRAVYYVVMQCLGAVCGAGVVKA
ZmPIP2;1                   SLVRALLYIVAQCLGAICGVGLVKA
ZmPIP2;2                   SLVRALLYMVAQCLGAVCGVGLVKA
ZmPIP2;3                   SLVRALLYIIAQCLGAICGVGLVKG
ZmPIP2;4                   SLVRALLYIIAQCLGAICGVGLVKG
ZmPIP2;5                   SLVRALLYIVAQCLGAICGVGLVKG
ZmPIP2;6                   SLVRALLYMAAQSLGAICGVALVKG
ZmPIP2;7                   SLVRALLYMIAQCAGAICGAGLAKG
AtPIP1;1                   SLTRALYYIVMQCLGAICGAGVVKG
AtPIP1;2                   SLTRAVYYIVMQCLGAICGAGVVKG
AtPIP1;3                   SLTRAVFYIVMQCLGAICGAGVVKG
AtPIP1;4                   SLTRAVFYMIMQCLGAICGAGVVKG
AtPIP1;5                   SLTRALFYIVMQCLGAICGAGVVKG
AtPIP2;1                   SLPRALLYIIAQCLGAICGVGFVKA
AtPIP2;2                   SLIRAVLYMVAQCLGAICGVGFVKA
AtPIP2;3                   SLIRAVLYMVAQCLGAICGVGFVKA
AtPIP2;4                   SLVRTVLYIVAQCLGAICGCGFVKA
AtPIP2;5                   TLVRAVMYMVAQCLGAICGVALVKA
AtPIP2;6                   SLVRAVSYMVAQCLGATCGVGLVKV
AtPIP2;7                   SLVRALGYMIAQCLGAICGVGFVKA
AtPIP2;8                   SLPRAVAYMVAQCLGAICGVGLVKA
```

Helix 3 TIP

```
1J4N          92 SVLRAIMYIIAQCVGAIVATAILSG 116
1FX8          82 DKRKVIPFIVSQVAGAFCAAALVYG 106
1RC2          77 PAKEVVGYVIAQVVGGIVAAALLYL 101
1Z98         115 SLLRALVYMIAQCLGAICGVGLVKA 139
2B6O          82 SLLRAICYVVAQLLGAVAGAAVLYS 106
2F2B          96 PGREVVPYIIAQLLGAAFGSFIFLQ 120
OsTIP1;1         TLFRGLLYWIAQLLGSTVACFLLRF
OsTIP1;2         SLVKAVVYWVAQLLGSVVACLLLKI
OsTIP2;1         TILTGLFYWIAQLLGASIACLLLKF
OsTIP2;2         TILTGVFYWIAQLLGAIVGAVLVQF
OsTIP2;3         TILTGIFYWVAQLLGASVACLLCSS
OsTIP3;1         SLIRALFYWLAQLLGAVVATLLLRL
OsTIP3;2         SFARAALYWAAQLLGAVLAVLLLRL
OsTIP4;1         TAFRSALYVAAQLLASSLACILLRY
OsTIP4;2         TLFRSALYVAAQLAGSSLACLLLRC
OsTIP4;3         TLFRSALYAAAQLLGSSLACLLLAA
OsTIP5;1         TVPSAIFYWASQMLGSTFACLVLHY
ZmTIP1;1         TLFRGLLYWVAQLLGSTVACFLLRF
ZmTIP1;2         SLLKALVYWVAQLLGSVVACLLLKI
ZmTIP2;1         TILTGVFYWVAQLLGATVACLLLGF
ZmTIP2;2         TVLTGLFYWVAQLLGASVACLLLRF
ZmTIP2;3         TILTGILYWVAQLLGASVACFLLQY
ZmTIP3;1         SLVRAVLYWVAQLLGAVAATLLLRL
ZmTIP4;1         TKLRAVLYVAAQLLASSAACVLLRF
ZmTIP4;2         TKLRALLYVAAQLLASSLACILLRY
ZmTIP4;3         TLFRSSLYIAAQMLASSAACFLLRW
ZmTIP4;4         TLFRSALYVAAQLLGSTLACLLLAF
ZmTIP5;1         GVPSAMFYWASQLLGATFACLSLNL
AtTIP1;1         TLLRGILYWIAQLLGSVVACLILKF
AtTIP1;2         TLLRGILYWIAQLLGSVAACFLLSF
AtTIP1;3         TLLRAILYWIAQLLGAVVACLLLKV
AtTIP2;1         TVITGVFYWIAQLLGSTAACFLLKY
AtTIP2;2         TVITGFFYWIAQCLGSIVACLLLVF
AtTIP2;3         TLITGFFYWIAQCLGSIVACLLLVF
AtTIP3;1         TAIRAIYYWIAQLLGAILACLLLRL
AtTIP3;2         SVIRAIYYWVAQLIGAILACLLLRL
AtTIP4;1         SVFRAFLYWIDQLLASSAACFLLSY
AtTIP5;1         SVPTAMFYWTSQMIASVMACLVLKV
```

Helix 3 NIP

1J4N	92	SVLRAIMYIIAQCVGAIVATAILSG	116
1FX8	82	DKRKVIPFIVSQVAGAFCAAALVYG	106
1RC2	77	PAKEVVGYVIAQVVGGIVAAALLYL	101
1Z98	115	SLLRALVYMIAQCLGAICGVGLVKA	139
2B6O	82	SLLRAICYVVAQLLGAVAGAAVLYS	106
2F2B	96	PGREVVPYIIAQLLGAAFGSFIFLQ	120
OsNIP1;1		PWRQVPAYAAAQMLGATLAAGTLRL	
OsNIP1;2		RWKQVPSYVVAQVLGSTMASLTLRV	
OsNIP1;3		PWRRVPAYAAAQVAGSAAASAALRA	
OsNIP1;4		PWRRAPAYALAQTAAATAASVVLRL	
OsNIP1;5		VVINVPSYVVAQVLGSTMASLTLRV	
OsNIP2;1		PWIQVPFYWAAQFTGAICASFVLKA	
OsNIP2;2		PWIQVPFYWAAQFTGAMCAAFVLRA	
OsNIP3;1		PWLQVPAYVAVQVLGSICAGFALKG	
OsNIP3;2		PPAHLLPYIAAQILGSITASFAVKG	
OsNIP3;3		PSAHLLPYISSQILGAVAASFAVKG	
OsNIP3;4		QPAHLLPYAAAQVLGAVAAAAAVDG	
OsNIP3;5		PPAHLLPYAAVQTAASLAAAFLAKG	
OsNIP4;1		VWPKLPLYVAAQLAGSLLACLSVNA	
ZmNIP1;1		PWRQLPAYVLAQMLGATLASGTLRL	
ZmNIP2;1		PWIQVPFYWAAQFTGSICASFVLKA	
ZmNIP2;2		PWIQVPFYWAAQFTGAMCAAFVLKA	
ZmNIP3;1		PWLQVPAYVAVQALASVCAAFALKG	
AtNIP1;1		PLKQVPAYVISQVIGSTLAAATLRL	
AtNIP1;2		PLKQVPAYVISQVIGSTLAAATLRL	
AtNIP2;1		PLNQVPAYITVQVIGSTLASATLRL	
AtNIP3;1		PFNQVPGYIAAQLLGSTLAAAVLRL	
AtNIP4;1		PWHQVPLYIGAQFAGSLLASLTLRL	
AtNIP4;2		PWYQVPLYIGAQLTGSLLASLTLRL	
AtNIP5;1		PWAHVPAYIAAQVSASICASFALKG	
AtNIP6;1		PWKHVPVYIGAQVMASVSAAFALKA	
AtNIP7;1		PWSQVPLYITAQTLGATAATLVGVS	

Helix 3 SIP

1J4N	92	SVLRAIMYIIAQCVGAIVATAILSG	116
1FX8	82	DKRKVIPFIVSQVAGAFCAAALVYG	106
1RC2	77	PAKEVVGYVIAQVVGGIVAAALLYL	101
1Z98	115	SLLRALVYMIAQCLGAICGVGLVKA	139
2B6O	82	SLLRAICYVVAQLLGAVAGAAVLYS	106
2F2B	96	PGREVVPYIIAQLLGAAFGSFIFLQ	120
OsSIP1;1		SLFPLALRFPAQAAGAVGGAMAISE	
OsSIP2;1		YLFAAFVRMPAQVFGSILGVKLIRA	
ZmSIP1;1		SLFSVALRFPAQAAGAVGGALAISE	
ZmSIP1;2		SLFSIALRLPAQAAGAVGGALAISE	
ZmSIP2;1		YLFTAFARIPAQVIGAVLGVKLIQV	
AtSIP1;1		TLFSLAIRLPAQAIGAAGGALAIME	
AtSIP1;2		SLFSLAIRSPAQAIGAAGGAITIME	
AtSIP2;1		FIFSVFVRIPVEVIGSILAVKHIIH	
