# Supplementary material for: Homology modeling of major intrinsic proteins in rice, maize and Arabidopsis: comparative analysis of transmembrane helix association and aromatic/arginine selectivity filters
Source: BMC Struct Biol. 2007 Apr 19;7:27. doi: 10.1186/1472-6807-7-27 (PMC1866351; doi:10.1186/1472-6807-7-27)
Supplement: Additional File 4 — Structure-based sequence alignment of plant MIPs in TM4 region. Structure-based sequence alignments are provided for all the 105 plant MIPs from the three plant species in the TM4 region. The first six sequences correspond to the experimentally determined aquaporin structures from different species. Their respective PDB IDs are shown in the first column. The beginning and end residue numbers are also given for each PDB structure. Small and weakly polar residues (Gly, Ala, Thr, Ser and Cys) occurring in the helix-helix interfaces are shaded in gray color. [file 1472-6807-7-27-S4.pdf]

Structure-based sequence alignment of TM4

Helix 4 PIP

1J4N	137	SGQGLGIEIIGTLQLVLCVLATT	159
1FX8	145	FVQAFAVEMVITAILMGLILALT	167
1RC2	131	MLSALVVELVLSAGFLLVIHGAT	153
1Z98	161	KGTALGAEIIGTFVLVYTVFSAT	183
2B6O	127	VGQATIVEIFLTLQFVLCIFATY	149
2F2B	143	YWQAMLAEVVGTFLLMITIMGIA	165
OsPIP1;1		KGDGLGAEIVGTFILVYTVFSAT	
OsPIP1;2		KGDGLGAEIVGTFILVYTVFSAT	
OsPIP1;3		KGDGLGAEIVGTFVLVYTVFSAT	
OsPIP1;4		KGDGLGAEIVGTFILVYTVFSAT	
OsPIP1;5		KGDRLGAEIVGTFILVYTVFSAT	
OsPIP2;1		KGTGLAAEIIGTFVLVYTVFSAT	
OsPIP2;2		KGTGLGAEIIGTFVLVYTVFSAT	
OsPIP2;3		KGTGLAAEIIGTFVLVYTVFSAT	
OsPIP2;4		TGTGLAAEIIGTFVLVYTVFSAT	
OsPIP2;5		KGTGLAAEIIGTFVLVYTVFSAT	
OsPIP2;6		TGTALGAEIIGTFVLVYTVFSAT	
OsPIP2;7		AAGALGAEIVGTFILVYTVFSAT	
OsPIP2;8		AGAGVVAEMVGTFVLVYTVFSAT	
ZmPIP1;1		KGDGLGAEIVGTFILVYTVFSAT	
ZmPIP1;2		KGDGLGAEIVGTFILVYTVFSAT	
ZmPIP1;3		KGDGLGAEIVGTFILVYTVFSAT	
ZmPIP1;4		KGDGLGAEIVGTFILVYTVFSAT	
ZmPIP1;5		KGDGLGAEIVGTFVLVYTVFSAT	
ZmPIP1;6		KGDGLGAEVVGTFVLVYTVFSAT	
ZmPIP2;1		RGTGLGAEIIGTFVLVYTVFSAT	
ZmPIP2;2		RGAGLGAEIVGTFVLVYTVFSAT	
ZmPIP2;3		KGTGLAAEIIGTFVLVYTVFSAT	
ZmPIP2;4		KGTGLAAEIIGTFVLVYTVFSAT	
ZmPIP2;5		KGTGLAAEIIGTFVLVYTVFSAT	
ZmPIP2;6		TGTGLAAEIIGTFVLVYTVFSAT	
ZmPIP2;7		KGTALGAEIIGTFVLVYTVFSAT	
AtPIP1;1		KGSGLGAEIIGTFVLVYTVFSAT	
AtPIP1;2		KGSGLGAEIIGTFVLVYTVFSAT	
AtPIP1;3		KGSGLGAEIIGTFVLVYTVFSAT	
AtPIP1;4		KGSGLGAEIIGTFVLVYTVFSAT	
AtPIP1;5		KGSGLGAEIVGTFVLVYTVFSAT	
AtPIP2;1		TGTGLAAEIIGTFVLVYTVFSAT	
AtPIP2;2		TGTGLAAEIIGTFVLVYTVFSAT	
AtPIP2;3		TGTGLAAEIIGTFVLVYTVFSAT	
AtPIP2;4		KGTGLGAEIIGTFVLVYTVFSAT	
AtPIP2;5		IGTGVAAEIIGTFVLVYTVFSAT	
AtPIP2;6		VGVGVGAEIIGTFVLVYTVFSAT	
AtPIP2;7		KGTALGAEIIGTFVLVYTVFSAT	
AtPIP2;8		TGTALGAEIIGTFVLVYTVFSAT	

Helix4 TIP

```
1J4N        137 SGQGLGIEIIGTLQLVLCVLATT 159
1FX8        145 FVQAFAVEMVITAILMGLILALT 167
1RC2        131 MLSALVVELVLSAGFLLVIHGAT 153
1Z98        161 KGTALGAEIIGTFVLVYTVFSAT 183
2B6O        127 VGQATIVEIFLTLQFVLCIFATY 149
2F2B        143 YWQAMLAEVVGTFLLMITIMGIA 165
OsTIP1;1        VWEALVLEIVMTFGLVYTVYATA
OsTIP1;2        AWNAVVFEIVMTFGLVYTVYATA
OsTIP2;1        ELEGVVMEIVITFALVYTVYATA
OsTIP2;2        AFEGVVMEIIVTFGLVYTVYATA
OsTIP2;3        EIEGMVMEIVITFALVYTVYATA
OsTIP3;1        DWHAVLLEATMTFGLMYAYYATV
OsTIP3;2        ERHALLLEVVMTFGLVYTVYATA
OsTIP4;1        PMQGLVMEIILTFSLLFVVYATI
OsTIP4;2        PVQGVAAEAVFTFTLLLVICATI
OsTIP4;3        AARAVAMEAVLTFSLLFAVYATV
OsTIP5;1        GFGAGILEGVLTFMVVYTVHVAG
ZmTIP1;1        VWEALVLEIVMTFGLVYTVYATA
ZmTIP1;2        AMNAVVLEMVMTFGLVYTVYATA
ZmTIP2;1        ELEGVVFEVVITFALVYTVYATA
ZmTIP2;2        ELEGVVFEIVITFALVYTVYATA
ZmTIP2;3        EIEGVVMEIVITFALVYTVYATA
ZmTIP3;1        DWHAVLLEAVMTFGLMYAYYATV
ZmTIP4;1        PMQGLVMEVILTFSLLFVTYAMI
ZmTIP4;2        PMQGLVMEVILTFSLLFVTYAMI
ZmTIP4;3        PLQGVVAEAVFTFSLLFVIYATI
ZmTIP4;4        ALRGVLMEAVLTFSLLFAVYATV
ZmTIP5;1        GFGGAVLEGVLTFLLVYTVHVVG
AtTIP1;1        VLNAFVFEIVMTFGLVYTVYATA
AtTIP1;2        SLNALVFEIVMTFGLVYTVYATA
AtTIP1;3        PWNAVVFEIVMTFGLVYTVYATA
AtTIP2;1        SIEGVVMEIIITFALVYTVYATA
AtTIP2;2        AIEGVVMEIVVTFALVYTVYATA
AtTIP2;3        AVEGVVMEIVVTFALVYTVYATA
AtTIP3;1        AVNGLVLEIILTFGLVYVVYSTL
AtTIP3;2        ELHGLLMEIILTFALVYVVYSTA
AtTIP4;1        YTQGIIWEIILTFSLLFTVYATI
AtTIP5;1        GFGASVLEGVLAFVLVYTVFTAS
```

Helix 4 NIP

```
1J4N                    137 SGQGLGIEIIGTLQLVLCVLATT 159
1FX8                    145 FVQAFAVEMVITAILMGLILALT 167
1RC2                    131 MLSALVVELVLSAGFLLVIHGAT 153
1Z98                    161 KGTALGAEIIGTFVLVYTVFSAT 183
2B6O                    127 VGQATIVEIFLTLQFVLCIFATY 149
2F2B                    143 YWQAMLAEVVGTFLLMITIMGIA 165
OsNIP1;1                    DVQSLVLEFIITFYLMFVISGVA
OsNIP1;2                    MAQAAALEFVISFFLMFVVSGVA
OsNIP1;3                    DVQSLAMEFIITFYLMFVVSGVA
OsNIP1;4                    HAQSLVIEFVITFYLMFVIMAVA
OsNIP1;5                    MAQAAALEFVISFFLMFVVSSVA
OsNIP2;1                    HWHSLVVEVIVTFNMMFVTLAVA
OsNIP2;2                    HWHALVIEIVVTFNMMFVTCAVA
OsNIP3;1                    TAQAFFTEFIITFNLLFVVTAVA
OsNIP3;2                    TVEAFFLEFVTTFVLLFIITALA
OsNIP3;3                    TVEAFFVEFIITFFLLFIITALA
OsNIP3;4                    VKELIAVAVGGTAMMNVLVAG--
OsNIP3;5                    AGEAFVVEVALTFVLSKELVAI-
OsNIP4;1                    TRLPFLMEFLASAVLMIVIATVA
ZmNIP1;1                    EVQSLVIEIITTFYLMFVISGVA
ZmNIP2;1                    HWHSLVIEIIVTFNMMFVTLAVA
ZmNIP2;2                    HWHALLIEIVVTFNMMFVTCAVA
ZmNIP3;1                    TAQAFFTEFIISFNLLFVVTAVA
AtNIP1;1                    DLQAFTMEFIVTFYLMFIISGVA
AtNIP1;2                    NLQSFVIEFIITFYLMFVISGVA
AtNIP2;1                    DLQAFVMEFIITGFLMLVVCAVT
AtNIP3;1                    NTTSFVMEFIATFNLMFVISAVA
AtNIP4;1                    PARALVAEIIISFLLMFVISGVA
AtNIP4;2                    SGQALVAEIIISFLLMFVISGVA
AtNIP5;1                    LGQAFALEFIITFILLFVVTAVA
AtNIP6;1                    LSQAFALEFIISFNLMFVVTAVA
AtNIP7;1                    CVSAFFVELIATSIVVFLASALH
```

Helix 4 SIP

```
1J4N                    137 SGQGLGIEIIGTLQLVLCVLATT 159
1FX8                    145 FVQAFAVEMVITAILMGLILALT 167
1RC2                    131 MLSALVVELVLSAGFLLVIHGAT 153
1Z98                    161 KGTALGAEIIGTFVLVYTVFSAT 183
2B6O                    127 VGQATIVEIFLTLQFVLCIFATY 149
2F2B                    143 YWQAMLAEVVGTFLLMITIMGIA 165
OsSIP1;1                    LHTGAAAELVLTFVITLAVLWII
OsSIP2;1                    VHHGALAEGLATFMVVIVSVTLK
ZmSIP1;1                    PHTGALAEGVLTFVITLTVLWVI
ZmSIP1;2                    PHTGAGAELVLTFVITLAVLLII
ZmSIP2;1                    AHHGALAEGLATFMVVMVSVTLK
AtSIP1;1                    VHTGAIAETILSFGITFAVLLII
AtSIP1;2                    AHNGAISEVVLSFSVTFLVLLII
AtSIP2;1                    IHHGALTEGILTFFIVLLSMGLT
```
