# Supplementary material for: Homology modeling of major intrinsic proteins in rice, maize and Arabidopsis: comparative analysis of transmembrane helix association and aromatic/arginine selectivity filters
Source: BMC Struct Biol. 2007 Apr 19;7:27. doi: 10.1186/1472-6807-7-27 (PMC1866351; doi:10.1186/1472-6807-7-27)
Supplement: Additional File 5 — Structure-based sequence alignment of plant MIPs in TM5 region. Structure-based sequence alignments are provided for all the 105 plant MIPs from the three plant species in the TM5 region. The first six sequences correspond to the experimentally determined aquaporin structures from different species. Their respective PDB IDs are shown in the first column. The beginning and end residue numbers are also given for each PDB structure. Small and weakly polar residues (Gly, Ala, Thr, Ser and Cys) occurring in the helix-helix interfaces are shaded in gray color. The residues forming arginine/aromatic selectivity filter are shown in bold. [file 1472-6807-7-27-S5.pdf]

Structure-based sequence alignment of TM5

Helix 5 PIP

1J4N	169	SGPLAIGFSVALG**H**LLAID	187
1FX8	178	LAPLLIGLLIAVI**G**ASMGP	196
1RC2	161	FAPIAIGLALTLI**H**LISIP	179
1Z98	197	LAPLPIGFAVFMV**H**LATIP	215
2B6O	159	SVALAVGFSLTLG**H**LFGMY	177
2F2B	174	FAGIIIGLTVAGI**I**TTLGN	192
OsPIP1;1		LAPLPIGFAVFLV**H**LATIP	
OsPIP1;2		LAPLPIGFAVFLV**H**LATIP	
OsPIP1;3		LAPLPIGFAVFLV**H**LATIP	
OsPIP1;4		LAPLPIGFAVFLV**H**LATIP	
OsPIP1;5		LAPLPIGFAVFLV**H**LATIP	
OsPIP2;1		LAPLPIGFAVFMV**H**LATIP	
OsPIP2;2		LAPLPIGFAVFMV**H**LATIP	
OsPIP2;3		LAPLPIGFAVFMV**H**LATIP	
OsPIP2;4		LAPLPIGFAVFMV**H**LATIP	
OsPIP2;5		LAPLPIGFAVFMV**H**LATIP	
OsPIP2;6		LAPLPIGFAVFMV**H**LATIP	
OsPIP2;7		LVPLPIGFAVFVV**H**LATIP	
OsPIP2;8		LAPLPIGLAVLVV**H**LATIP	
ZmPIP1;1		LAPLPIGFAVFLV**H**LATMG	
ZmPIP1;2		LAPLPIGFAVFLV**H**LATIP	
ZmPIP1;3		LAPLPIGFAVFLV**H**LATIP	
ZmPIP1;4		LAPLPIGFAVFLV**H**LATIP	
ZmPIP1;5		LAPLPIGFAVFLV**H**LATIP	
ZmPIP1;6		LAPLPIGFAVFLV**H**LATIP	
ZmPIP2;1		LAPLPIGFAVFMV**H**LATIP	
ZmPIP2;2		LAPLPIGFAVFMV**H**LATIP	
ZmPIP2;3		LAPLPIGFAVFMV**H**LATIP	
ZmPIP2;4		LAPLPIGFAVFMV**H**LATIP	
ZmPIP2;5		LAPLPIGFAVFMV**H**LATIP	
ZmPIP2;6		LAPLPIGFAVFMV**H**LATIP	
ZmPIP2;7		LAPLPIGFAVFMV**H**LATIP	
AtPIP1;1		LAPLPIGFAVFLV**H**LATIP	
AtPIP1;2		LAPLPIGFAVFLV**H**LATIP	
AtPIP1;3		LAPLPIGFAVFLV**H**LATIP	
AtPIP1;4		LAPLPIGFAVFLV**H**LATIP	
AtPIP1;5		LAPLPIGFAVFLV**H**LATIP	
AtPIP2;1		LAPLPIGFAVFMV**H**LATIP	
AtPIP2;2		LAPLPIGFAVFMV**H**LATIP	
AtPIP2;3		LAPLPIGFAVFMV**H**LATIP	
AtPIP2;4		LAPLPIGFAVFMV**H**LATIP	
AtPIP2;5		LAPLPIGFAVFIV**H**LATIP	
AtPIP2;6		LAPLPIGFSVFMV**H**LATIP	
AtPIP2;7		LAPLPIGFAVFMV**H**LATIP	
AtPIP2;8		LAPLPIGFAVFMV**H**LATIP	

Helix 5 TIP

1J4N	169	SGPLAIGFSVALG**H**LLAID	187
1FX8	178	LAPLLIGLLIAVI**G**ASMGP	196
1RC2	161	FAPIAIGLALTLI**H**LISIP	179
1Z98	197	LAPLPIGFAVFMV**H**LATIP	215
2B6O	159	SVALAVGFSLTLG**H**LFGMY	177
2F2B	174	FAGIIIGLTVAGI**I**TTLGN	192
OsTIP1;1		IAPIAIGFIVGAN**I**LVGGA	
OsTIP1;2		IAPIAIGFIVGAN**I**LAGGA	
OsTIP2;1		IAPIAIGFIVGAN**I**LAAGP	
OsTIP2;2		IAPIAIGFIVGAN**I**LVAGP	
OsTIP2;3		VAPMAIGFIVGAN**I**LAAGP	
OsTIP3;1		IAPLAVGFLLGAN**M**LAGGP	
OsTIP3;2		IAPLAIGLVAGAN**I**LAGGP	
OsTIP4;1		FGPLLTGLIVGAN**T**IAGGN	
OsTIP4;2		TGPLLTGLLVGAN**T**VAGGA	
OsTIP4;3		LGPLLVGLVVGAN**I**LAGGP	
OsTIP5;1		LGALVVGAVTGAC**V**LAAGS	
ZmTIP1;1		IAPIAIGFIVGAN**I**LVGGA	
ZmTIP1;2		IAPIAIGFIVGAN**I**LAGGA	
ZmTIP2;1		IAPIAIGFIVGAN**I**LAAGP	
ZmTIP2;2		IAPIAIGFIVGAN**I**LAAGP	
ZmTIP2;3		IAPMAIGFIVGAN**I**LAAGP	
ZmTIP3;1		IAPLAVGFLLGAN**V**LAGGP	
ZmTIP4;1		IGPLLTGLIVGAN**S**LAGGN	
ZmTIP4;2		IGPLLTGLIVGAN**S**LAGGN	
ZmTIP4;3		AGPLLTGLLVGAN**S**VAGAA	
ZmTIP4;4		MGPLLVGLVVGAN**V**LAGGP	
ZmTIP5;1		LGALAVGLTQGAF**V**LAAGA	
AtTIP1;1		IAPIAIGFIVGAN**I**LAGGA	
AtTIP1;2		IAPIAIGFIVGAN**I**LAGGA	
AtTIP1;3		IAPLAIGLIVGAN**I**LVGGA	
AtTIP2;1		IAPLAIGLIVGAN**I**LAAGP	
AtTIP2;2		IAPIAIGFIVGAN**I**LAAGP	
AtTIP2;3		IAPIAIGFIVGAN**I**LAAGP	
AtTIP3;1		IAPLAIGLIVGAN**I**LVGGP	
AtTIP3;2		IAPLAIGLIVGAN**I**LVGGP	
AtTIP4;1		FGPLLTGFVVGAN**I**LAGGA	
AtTIP5;1		VGPIFIGFVAGAN**V**LAAGP	

Helix 5 NIP

1J4N	169	SGPLAIGFSVALG**H**LLAID	187
1FX8	178	LAPLLIGLLIAVI**G**ASMGP	196
1RC2	161	FAPIAIGLALTLI**H**LISIP	179
1Z98	197	LAPLPIGFAVFMV**H**LATIP	215
2B6O	159	SVALAVGFSLTLG**H**LFGMY	177
2F2B	174	FAGIIIGLTVAGI**I**TTLGN	192
OsNIP1;1		LAGLAVGATILLN**V**LIAGP	
OsNIP1;2		LAGLAVGATVAVN**V**LFAGP	
OsNIP1;3		LAGLAVGATVLVN**V**LFAGP	
OsNIP1;4		MAGVAVGGTIMLN**V**LFAGP	
OsNIP1;5		LAGLAVGATVAVN**V**LFAGP	
OsNIP2;1		LAGLAVGSAVCIT**S**IFAGA	
OsNIP2;2		LAGLAVGSAVCIT**S**IFAGP	
OsNIP3;1		LAGIAVGAAVTLN**I**LIAGP	
OsNIP3;2		LIAVAVGATIMMN**A**LVAGP	
OsNIP3;3		LIAVAVGATVMMN**I**LVAGP	
OsNIP3;4		--YACEILLCIYN**V**LVAGP	
OsNIP3;5		----AIAAAIMMN**A**LVGGP	
OsNIP4;1		VGGIAIGAAVGGL**G**LVIGP	
ZmNIP1;1		LAGLAVGATILLN**V**LIAGP	
ZmNIP2;1		LAGLAVGSAVCIT**S**IFAGA	
ZmNIP2;2		LAGLAVGSAVCIT**S**IFAGP	
ZmNIP3;1		LAGIAVGAAVTLN**I**LVAGP	
AtNIP1;1		LAGLAIGSTVLLN**V**LIAAP	
AtNIP1;2		LAGLAVGSTVLLN**V**IIAGP	
AtNIP2;1		LEGLIIGATVTLN**V**IFAGE	
AtNIP3;1		FAGIAIGATIVLD**I**LFSGP	
AtNIP4;1		LAGIAVGMTIMVN**V**FVAGP	
AtNIP4;2		LAGIAVGMTIILN**V**FVAGP	
AtNIP5;1		LAGIAVGATVMLN**I**LVAGP	
AtNIP6;1		LAGIAVGATVMLN**I**LIAGP	
AtNIP7;1		LTGFVIGTVISLG**V**LITGP	

Helix 5 SIP

1J4N	169	SGPLAIGFSVALG**H**LLAID	187
1FX8	178	LAPLLIGLLIAVI**G**ASMGP	196
1RC2	161	FAPIAIGLALTLI**H**LISIP	179
1Z98	197	LAPLPIGFAVFMV**H**LATIP	215
2B6O	159	SVALAVGFSLTLG**H**LFGMY	177
2F2B	174	FAGIIIGLTVAGI**I**TTLGN	192
OsSIP1;1		VKTWMLSISTVCL**V**LTGAA	
OsSIP2;1		MKTWISSIWKMTF**H**LLSSD	
ZmSIP1;1		LKTLLLSTSIVSV**I**LAGAE	
ZmSIP1;2		IKTWMISICTLCL**V**LSGAA	
ZmSIP2;1		MKTWITSIWKNTI**H**LLSSD	
AtSIP1;1		AKTFLLALATISF**V**VAGSK	
AtSIP1;2		AKTFLLALATVSV**F**VVGSK	
AtSIP2;1		MKTWIGSLAKLTL**H**ILGSD	
