# Supplementary material for: Homology modeling of major intrinsic proteins in rice, maize and Arabidopsis: comparative analysis of transmembrane helix association and aromatic/arginine selectivity filters
Source: BMC Struct Biol. 2007 Apr 19;7:27. doi: 10.1186/1472-6807-7-27 (PMC1866351; doi:10.1186/1472-6807-7-27)
Supplement: Additional File 6 — Structure-based sequence alignment of plant MIPs in TM6 region. Structure-based sequence alignments are provided for all the 105 plant MIPs from the three plant species in the TM6 region. The first six sequences correspond to the experimentally determined aquaporin structures from different species. Their respective PDB IDs are shown in the first column. The beginning and end residue numbers are also given for each PDB structure. Small and weakly polar residues (Gly, Ala, Thr, Ser and Cys) occurring in the helix-helix interfaces are shaded in gray color. [file 1472-6807-7-27-S6.pdf]

Structure-based sequence alignment of TM6

```
Helix 6  PIP

1J4N                     212 WIFWVGPFIGAALAVLIYDFI 232
1FX8                     234 LVPLFGPIVGAIVGAFAYRKL 254
1RC2                     206 WFFWVVPIVGGIIGGLIYRTL 226
1Z98                     243 WIFWVGPFIGAAVAAAYHQYV 263
2B6O                     202 WVYWVGPVIGAGLGSLLYDFL 222
2F2B                     223 SIYVIGPIVGAVLAALTYQYL 243
OsPIP1;1                     WIFWVGPFVGAALAAIYHQVI
OsPIP1;2                     WIFWVGPFIGAALAAIYHQVV
OsPIP1;3                     WIFWVGPFIGAALAAIYHVVV
OsPIP1;4                     WIFWVGPFVGAALAAIYHQVI
OsPIP1;5                     WIFWVGPLMGGALVAIYHQGV
OsPIP2;1                     WIFWVGPFVGAAIAAFYHQYI
OsPIP2;2                     WIFWVGPLIGAAIAAAYHQYV
OsPIP2;3                     WIFWVGPLIGAAIAAAYHQYV
OsPIP2;4                     WIFWVGPFIGAAIAALYHQVI
OsPIP2;5                     WIFWVGPFIGAAIAALYHQIV
OsPIP2;6                     WIFWAGPFIGALAAAAYHQYI
OsPIP2;7                     WIFWVGPVIGAFLAAAYHKLV
OsPIP2;8                     WIFWVGPFAGAAAAMIYHHYI
ZmPIP1;1                     WIFWVGPFIGAALAAIYHQVI
ZmPIP1;2                     WIFWVGPFIGAALAAIYHQVI
ZmPIP1;3                     WIFWVGPFIGAALAAIYHQVI
ZmPIP1;4                     WIFWVGPFIGAALAAIYHQVI
ZmPIP1;5                     WIFWVGPFIGAALAAIYHVVI
ZmPIP1;6                     WIFWVGPFAGAALAAVYHQVV
ZmPIP2;1                     WIFWVGPLVGAAIAAFYHQYI
ZmPIP2;2                     WIFWVGPLLGAAIAAFYHQYI
ZmPIP2;3                     WIFWVGPLIGAAIAAAYHQYV
ZmPIP2;4                     WIFWVGPLIGAAIAAAYHQYV
ZmPIP2;5                     WIFWVGPFIGAAIAAAYHQYV
ZmPIP2;6                     WIFWVGPFIGAAIAALYHQIV
ZmPIP2;7                     WIYWVGPFVGAAVAAIYHQYI
AtPIP1;1                     WVFWVGPFIGAALAALYHVVV
AtPIP1;2                     WVFWVGPFIGAALAALYHVIV
AtPIP1;3                     WIFWVGPFIGAALAALYHQLV
AtPIP1;4                     WIFWVGPFIGAALAALYHQIV
AtPIP1;5                     WIFWVGPFIGAALAALYHQIV
AtPIP2;1                     WIFWVGPFIGAAIAAFYHQFV
AtPIP2;2                     WIFWVGPFIGAAIAAFYHQFV
AtPIP2;3                     WIFWVGPFIGATIAAFYHQFV
AtPIP2;4                     WIFWVGPMIGAAAAAFYHQFI
AtPIP2;5                     WIFWVGPFAGAAIAAFYHQFV
AtPIP2;6                     WIFWVGPFVGAAIAAFYHQFV
AtPIP2;7                     WIFWVGPFLGALAAAAYHQYI
AtPIP2;8                     WIFWVGPFVGALAAAAYHQYI
```

Helix 6 TIP

1J4N	212	WIFWVGPFIGAALAVLIYDFI	232
1FX8	234	LVPLFGPIVGAIVGAFAYRKL	254
1RC2	206	WFFWVVPIVGGIIGGLIYRTL	226
1Z98	243	WIFWVGPFIGAAVAAAYHQYV	263
2B6O	202	WVYWVGPVIGAGLGSLLYDFL	222
2F2B	223	SIYVIGPIVGAVLAALTYQYL	243
OsTIP1;1		WVYWVGPLIGGGLAGVIYEVL	
OsTIP1;2		WVYWLGPFVGAAIAALIYDII	
OsTIP2;1		WVYWVGPLIGGGLAGLVYGDV	
OsTIP2;2		WIYWVGPLVGGGLAGLVYRYV	
OsTIP2;3		WVYWVGPLIGGGLAGLVYDDV	
OsTIP3;1		WVYWLGPFVGAGLAGLLYEYL	
OsTIP3;2		WVYWLGPLIGAGMAGALYEFV	
OsTIP4;1		WIYWLGPLIGGPLAGLVYESL	
OsTIP4;2		WVYWVGPLAGGPLAVVAYELL	
OsTIP4;3		WIYWVGPLIGGPLAGLVYEGL	
OsTIP5;1		AVYWAGPMVGAAVAALVHQAL	
ZmTIP1;1		WVYWVGPLIGGGLAGVIYELL	
ZmTIP1;2		WVYWVGPLAGAAIAALVYDII	
ZmTIP2;1		WVYWVGPLVGGGLAGLVYGDV	
ZmTIP2;2		WVYWVGPLIGGGLAGLVYGDV	
ZmTIP2;3		WVYWVGPLVGGGLAGLVYGDV	
ZmTIP3;1		WVYWLGPFLGAGLAGLVYEYL	
ZmTIP4;1		WVYWIGPLLGGPLAGFVYESL	
ZmTIP4;2		WVYWIGPLLGGSLAGFVYESL	
ZmTIP4;3		WVYWVGPLAGGPLAVLVYECC	
ZmTIP4;4		WVYWVGPLIGGPLAGLVYDGL	
ZmTIP5;1		AVYWAGPMVGAAVAALVYQIM	
AtTIP1;1		WVYWAGPLVGGGIAGLIYEVF	
AtTIP1;2		WVYWAGPLIGGGLAGIIYDFV	
AtTIP1;3		WVYWVGPFIGAAIAAIVYDTI	
AtTIP2;1		WVYWVGPLIGGGLAGLIYGNV	
AtTIP2;2		WIYWVGPLVGGALAGLIYGDV	
AtTIP2;3		WIYWVGPLVGGALAGLIYGDV	
AtTIP3;1		WIYWVGPFIGSALAALIYEYM	
AtTIP3;2		WIYWVGPFIGGALAALIYEYM	
AtTIP4;1		WVYWVGPLIGGGLAGFIYENV	
AtTIP5;1		AVYWVGPLLGGATAALVYDNV	

Helix 6 NIP

1J4N	212	WIFWVGPFIGAALAVLIYDFI	232
1FX8	234	LVPLFGPIVGAIVGAFAYRKL	254
1RC2	206	WFFWVVPIVGGIIGGLIYRTL	226
1Z98	243	WIFWVGPFIGAAVAAAYHQYV	263
2B6O	202	WVYWVGPVIGAGLGSLLYDFL	222
2F2B	223	SIYVIGPIVGAVLAALTYQYL	243
OsNIP1;1		WVYIVGPVAGAVAGAWAYNII	
OsNIP1;2		WVYVAAPVSGTVCGAWAYNLL	
OsNIP1;3		WVYIAGPVFGAVAGAWAYNLI	
OsNIP1;4		WVYILGPFAGAAAGAWAYSLI	
OsNIP1;5		WVYVAAPVSGAVCGAWAYNLL	
OsNIP2;1		WIYFLGPVMGTLSGAWTYTFI	
OsNIP2;2		WIYFLGPVVGTLSGAWVYTYI	
OsNIP3;1		WIYLIAPTLGAVAGAGVYTAV	
OsNIP3;2		WVYLVATPLGAVAGEGFYFAI	
OsNIP3;3		WVYLVATPLGAIAGTGAYVAI	
OsNIP3;4		WVYMVSTPLGAIAGTGAYFAI	
OsNIP3;5		WIYLVAPPLGAIAGAATYTLI	
OsNIP4;1		WIYVVAPVAGMLVGALCNRAV	
ZmNIP1;1		WVYVVGPVVGAVAGAWAYNLI	
ZmNIP2;1		WIYFLGPVLGTLSGAWTYTYI	
ZmNIP2;2		WIYFLGPVIGTLSGAWVYTYI	
ZmNIP3;1		WIYLLAPTLGALAGASVYKAV	
AtNIP1;1		WIYLVAPTLGAIAGAWVYNTV	
AtNIP1;2		WIYIVSPIVGAVSGAWVYNMV	
AtNIP2;1		WIYLLAPTLGAVSGALIHKML	
AtNIP3;1		WLYIVSPVIGALSGAWTYGLL	
AtNIP4;1		WVYIVGPVLGVISGGFVYNLI	
AtNIP4;2		WVYIVGPFVGIFAGGFVYNFM	
AtNIP5;1		WVYLVAPTLGAISGAAVYTGV	
AtNIP6;1		WVYLTAPILGALIGAGTYTIV	
AtNIP7;1		WIYMTAPVIGAIIGVLTYRSI	

Helix 6 SIP

1J4N	212	WIFWVGPFIGAALAVLIYDFI	232
1FX8	234	LVPLFGPIVGAIVGAFAYRKL	254
1RC2	206	WFFWVVPIVGGIIGGLIYRTL	226
1Z98	243	WIFWVGPFIGAAVAAAYHQYV	263
2B6O	202	WVYWVGPVIGAGLGSLLYDFL	222
2F2B	223	SIYVIGPIVGAVLAALTYQYL	243
OsSIP1;1		YVYWICPFVGAVLAAWVFRAV	
OsSIP2;1		LVYWLAPLQATLLGVWVVTLL	
ZmSIP1;1		YVYWICPFIGAMLAGWIFRVV	
ZmSIP1;2		YVYWICPFIGAILAAWIFRAM	
ZmSIP2;1		LVYWLAPLQATLLGVWAVTFF	
AtSIP1;1		YVYWISSFVGALSAALLFRSI	
AtSIP1;2		YVYWISSYTGAILSAMLFRII	
AtSIP2;1		LVYWLGPVKATLLAVWFFKVV	
