# Supplementary material for: Homology modeling of major intrinsic proteins in rice, maize and Arabidopsis: comparative analysis of transmembrane helix association and aromatic/arginine selectivity filters
Source: BMC Struct Biol. 2007 Apr 19;7:27. doi: 10.1186/1472-6807-7-27 (PMC1866351; doi:10.1186/1472-6807-7-27)
Supplement: Additional File 7 — Structure-based sequence alignment of plant MIPs in the loop B region. Structure-based sequence alignments are provided for all the 105 plant MIPs from the three plant species in the loop B region. The first six sequences correspond to the experimentally determined aquaporin structures from different species. Their respective PDB IDs are shown in the first column. The beginning and end residue numbers are also given for each PDB structure. Small and weakly polar residues (Gly, Ala, Thr, Ser and Cys) occurring in the helix-helix interfaces are shaded in gray color. [file 1472-6807-7-27-S7.pdf]

Structure-based sequence alignment of Loop B

LB PIP

1J4N	72	ISGAHLNPAVTLGLLLS	88
1FX8	62	VSGAHLNPAVTIALWLF	78
1RC2	57	ISGGHFNPAVTIGLWAG	73
1Z98	95	ISGGHINPAVTFGLFLA	111
2B6O	62	ISGAHVNPAVTFAFLVG	78
2F2B	76	ISGCHINPAVTIGLWSV	92
OsPIP1;1		ISGGHINPAVTFGLFLA	
OsPIP1;2		ISGGHINPAVTFGLFLA	
OsPIP1;3		ISGGHINPAVTFGLFLA	
OsPIP2;1		ISGGHINPAVTFGLFLA	
OsPIP2;2		ISGGHINPAVTFGLFLA	
OsPIP2;3		VSGGHINPAVTFGLFLA	
OsPIP2;4		VSGGHINPAVTLGLFLA	
OsPIP2;5		ISGGHINPAVTFGLFLA	
OsPIP2;6		ISGGHINPAVTFGLLLA	
OsPIP2;7		VSGGHINPAVTLGLFFG	
OsPIP2;8		ISGGHMNPAVTFAMVLA	
OsPIP1;4		ISGRHINPAVTFGLFLA	
OsPIP1;5		ISGGHINPAVTFGLFLA	
ZmPIP1;1		ISG-HINPAVTFGLFLA	
ZmPIP1;2		ISGGHINPAVTFGLFLA	
ZmPIP1;3		ISGGHINPAVTFGLFLA	
ZmPIP1;4		ISGGHINPAVTFGLFLA	
ZmPIP1;5		ISGGHINPAVTFGLFLA	
ZmPIP1;6		VSGGHINPAVTFGLLLA	
ZmPIP2;1		ISGGHINPAVTFGLFLA	
ZmPIP2;2		ISGGHINPAVTFGLFLA	
ZmPIP2;3		ISGGHINPAVTFGLFLA	
ZmPIP2;4		ISGGHINPAVTFGLFLA	
ZmPIP2;5		VSGGHINPAVTFGLFLA	
ZmPIP2;6		ISGGHINPAVTFGLFLA	
ZmPIP2;7		ISGGHINPAVTFGLFLG	
AtPIP1;1		ISGGHINPAVTFGLFLA	
AtPIP1;2		ISGGHINPAVTFGLFLA	
AtPIP1;3		ISGGHINPAVTFGLFLA	
AtPIP1;4		ISGGHINPAVTFGLFLA	
AtPIP1;5		ISGGHINPAVTFGLFLA	
AtPIP2;1		ISGGHINPAVTFGLFLA	
AtPIP2;2		ISGGHINPAVTFGLFLA	
AtPIP2;3		ISGGHINPAVTFGLFLA	
AtPIP2;4		ISGGHINPAVTVGLFLA	
AtPIP2;5		ISGGHINPAVTFGLLLA	
AtPIP2;6		ISGGHINPAVTFGLFLA	
AtPIP2;7		ISGGHINPAVTFGLFLA	
AtPIP2;8		ISGGHINPAVTFGLFLA	

LB TIP

1J4N	72	ISGAHLNPAVTLGLLLS	88
1FX8	62	VSGAHLNPAVTIALWLF	78
1RC2	57	ISGGHFNPAVTIGLWAG	73
1Z98	95	ISGGHINPAVTFGLFLA	111
2B6O	62	ISGAHVNPAVTFAFLVG	78
2F2B	76	ISGCHINPAVTIGLWSV	92
OsTIP1;1		ISGGHVNPAVTFGAFVG	
OsTIP1;2		ISGGHVNPAVTFGAFVG	
OsTIP2;1		ISGGHLNPAVTFGLAVG	
OsTIP2;2		ISGGHVNPAVTFGLALG	
OsTIP2;3		ISGGHLNPVVTFGLAVG	
OsTIP3;1		ISGGHVNPAITFGALLG	
OsTIP3;2		ASGGHVNPAVTFGVLVG	
OsTIP4;1		VSGGHLNPAVTVALLAR	
OsTIP4;2		VSGGHLNPAVTLSLAVG	
OsTIP4;3		VSGGHINPAVTLGLAAG	
OsTIP5;1		VSGGHVNPAVTFAYAIG	
ZmTIP1;1		ISGGHVNPAVTFGAFVG	
ZmTIP1;2		ISGGHVNPAVTFGAFVG	
ZmTIP2;1		ISGGHLNPAVTFGLAVG	
ZmTIP2;2		TSGGHLNPAVTFGLAVG	
ZmTIP2;3		ISGGHLNPAVTFGLAVG	
ZmTIP3;1		ISGGHVNPAVTFGALVG	
ZmTIP4;1		VSGGHLNPAVTVGLMVR	
ZmTIP4;2		VSGGHLNPAVTVGILVR	
ZmTIP4;3		ISGGHVNPAVTLSLAVG	
ZmTIP4;4		VSGGHINPAVTLGLAAT	
ZmTIP5;1		VSGGHVNPAVTFAYAIG	
AtTIP1;1		ISGGHVNPAVTFGAFIG	
AtTIP1;2		ISGGHVNPAVTFGVLLG	
AtTIP1;3		VSGGHVNPAVTFGAFIG	
AtTIP2;1		ISGGHVNPAVTFGLAVG	
AtTIP2;2		ISGGHLNPAVTLGLAVG	
AtTIP2;3		ISGGHLNPAVTLGLAIG	
AtTIP3;1		VSGGHVNPAVTFGALVG	
AtTIP3;2		VSGGHVNPAVTFAALIG	
AtTIP4;1		ISGGHLNPAVTLGLLLG	
AtTIP5;1		VSGGHVNPAVTFAMAVA	

LB NIP

```
1J4N        72 ISGAHLNPAVTLGLLLS 88
1FX8        62 VSGAHLNPAVTIALWLF 78
1RC2        57 ISGGHFNPAVTIGLWAG 73
1Z98        95 ISGGHINPAVTFGLFLA 111
2B6O        62 ISGAHVNPAVTFAFLVG 78
2F2B        76 ISGCHINPAVTIGLWSV 92
OsNIP1;1       ISGAHFNPAVTLAF-AT
OsNIP1;2       ISGAHFNPAVTVAFATC
OsNIP1;3       ISGAHLNPAVTLAF-AT
OsNIP1;4       VSGAHLNPAVTLGFAVA
OsNIP1;5       ISGTHLNPAVTVAFATR
OsNIP2;1       ISGAHMNPAVTLAFAVF
OsNIP2;2       ISGAHMNPAVTLSFAFF
OsNIP3;1       ISGAHLNPSLTIAFAAL
OsNIP3;2       ISGCHLNPAVSIAMTVF
OsNIP3;3       ISGCHLNPAISIAMAVF
OsNIP3;4       VSGAHINPAVSVAMAAF
OsNIP3;5       ISGSHLNPAVSLAMAAL
OsNIP4;1       LGPAHFNPAVTITFAAY
ZmNIP1;1       ISGAHFNPAVTLAFATS
ZmNIP2;1       ISGAHMNPAVTLAFAVF
ZmNIP2;2       ISGAHMNPAVTLSFACF
ZmNIP3;1       ISGAHLNPSLTIAFAAL
AtNIP1;1       ISGAHINPAVTIAFASC
AtNIP1;2       ISGAHFNPAVTIAF-AS
AtNIP2;1       LS-AHFNPAVTLALASS
AtNIP3;1       VSGAHFNPAVSIAFASS
AtNIP4;1       ISGAHFNPAVTVTFAIF
AtNIP4;2       ISGAHFNPAVTVTFAVF
AtNIP5;1       ISGAHLNPSLTIAFAAL
AtNIP6;1       ISGAHLNPAVTIAFAAL
AtNIP7;1       ISGAHLNPSITIAFAVF
```

LB SIP

```
1J4N        72 ISGAHLNPAVTLGLLLS 88
1FX8        62 VSGAHLNPAVTIALWLF 78
1RC2        57 ISGGHFNPAVTIGLWAG 73
1Z98        95 ISGGHINPAVTFGLFLA 111
2B6O        62 ISGAHVNPAVTFAFLVG 78
2F2B        76 ISGCHINPAVTIGLWSV 92
OsSIP1;1       LGGASFNPTALAAFHAA
OsSIP2;1       TGGASYNPLTVLAGALA
ZmSIP1;1       LGGASFNPTDFAASYAA
ZmSIP1;2       LGGASFNPTGVAAFYAA
ZmSIP2;1       SGGASYNPLTVLAAALA
AtSIP1;1       -GSASFNPTGSAAFYVA
AtSIP1;2       LGGASFNPCGNAAFYTA
AtSIP2;1       TKGGLYNPLTALAAGVS
```
