# Supplementary material for: Homology modeling of major intrinsic proteins in rice, maize and Arabidopsis: comparative analysis of transmembrane helix association and aromatic/arginine selectivity filters
Source: BMC Struct Biol. 2007 Apr 19;7:27. doi: 10.1186/1472-6807-7-27 (PMC1866351; doi:10.1186/1472-6807-7-27)
Supplement: Additional File 8 — Structure-based sequence alignment of plant MIPs in the loop E region. Structure-based sequence alignments are provided for all the 105 plant MIPs from the three plant species in the loop E region. The first six sequences correspond to the experimentally determined aquaporin structures from different species. Their respective PDB IDs are shown in the first column. The beginning and end residue numbers are also given for each PDB structure. Small and weakly polar residues (Gly, Ala, Thr, Ser and Cys) occurring in the helix-helix interfaces are shaded in gray color. The residues forming arginine/aromatic selectivity filter are shown in bold. [file 1472-6807-7-27-S8.pdf]

Structure-based sequence alignment of Loop E

LE PIP

1J4N	188	YTG**C**GINPA**R**SFGSSVIT	205
1FX8	197	LTG**F**AMNPA**R**DFGPKVFA	214
1RC2	180	VTN**T**SVNPA**R**STAVAI-F	196
1Z98	216	ITG**T**GINPA**R**SFGAAVIF	233
2B6O	178	YTG**A**GMNPA**R**SFAPAILT	195
2F2B	193	ISG**S**SLNPA**R**TFGPYLND	210
OsPIP1;1		ITG**T**GINPA**R**SLGAAIIY	
OsPIP1;2		ITG**T**GINPA**R**SLGAAIIY	
OsPIP1;3		ITG**T**GINPA**R**SLGAAIVY	
OsPIP2;1		ITG**T**GINPA**R**SIGAAVIF	
OsPIP2;2		ITG**T**GINPA**R**SLGTAVIY	
OsPIP2;3		ITG**T**GINPA**R**SLGAAVIY	
OsPIP2;4		ITG**T**GINPA**R**SLGVAVVY	
OsPIP2;5		VTG**T**GINPA**R**SLGAAVVY	
OsPIP2;6		ITG**T**GINPA**R**SIGAAVIY	
OsPIP2;7		ITG**T**GINPA**R**SLGAAVLY	
OsPIP2;8		ITG**T**GINPA**R**SLGPALVL	
OsPIP1;4		ITG**T**GINPA**R**SLGAAIIY	
OsPIP1;5		ITG**T**GINPR**R**TWGCHHLQ	
ZmPIP1;1		ITG**T**GINPA**R**SLGAAVIY	
ZmPIP1;2		ITG**T**GINPA**R**SLGAAIIY	
ZmPIP1;3		ITG**T**GINPA**R**SLGAAIIY	
ZmPIP1;4		ITG**T**GINPA**R**SLGAAIIY	
ZmPIP1;5		ITG**T**GINPA**R**SLGAAIVY	
ZmPIP1;6		ITG**T**GINPA**R**SLGAAIIY	
ZmPIP2;1		VTG**T**GINPA**R**SLGAAVIY	
ZmPIP2;2		VTG**T**GINPA**R**SLGAAVVY	
ZmPIP2;3		ITG**T**GINPA**R**SLGAAVIY	
ZmPIP2;4		ITG**T**GINPA**R**SLGAAVIY	
ZmPIP2;5		ITG**T**GINPA**R**SLGAAVIY	
ZmPIP2;6		ITG**T**GINPA**R**SLGAAVVY	
ZmPIP2;7		VTG**T**GINPA**R**SFGPAVIF	
AtPIP1;1		ITG**T**GINPA**R**SLGAAIIY	
AtPIP1;2		ITG**T**GINPA**R**SLGAAIIF	
AtPIP1;3		ITG**T**GINPA**R**SLGAAIIY	
AtPIP1;4		ITG**T**GINPA**R**SLGAAIIY	
AtPIP1;5		ITG**T**GINPA**R**SLGAAIIY	
AtPIP2;1		ITG**T**GINPA**R**SFGAAVIY	
AtPIP2;2		ITG**T**GINPA**R**SFGAAVIY	
AtPIP2;3		ITG**T**GINPA**R**SFGAAVIF	
AtPIP2;4		ITG**T**GINPA**R**SFGAAVIY	
AtPIP2;5		ITG**T**GINPA**R**SLGAAIIY	
AtPIP2;6		ITG**T**GINPA**R**SFGAAVIY	
AtPIP2;7		ITG**T**GINPA**R**SFGAAVIY	
AtPIP2;8		ITG**T**GINPA**R**SFGAAVIY	

LE   TIP

1J4N	188	YTG**C**GINPA**R**SFGSSVIT	205
1FX8	197	LTG**F**AMNPA**R**DFGPKVFA	214
1RC2	180	VTN**T**SVNPA**R**STAVAI-F	196
1Z98	216	ITG**T**GINPA**R**SFGAAVIF	233
2B6O	178	YTG**A**GMNPA**R**SFAPAILT	195
2F2B	193	ISG**S**SLNPA**R**TFGPYLND	210
OsTIP1;1		FDG**A**SMNPA**V**SFGPALVS	
OsTIP1;2		FDG**A**SMNPA**V**SFGPAVVT	
OsTIP2;1		FSG**G**SMNPA**R**SFGPAVAA	
OsTIP2;2		FSG**G**SMNPA**R**SFGPAVAS	
OsTIP2;3		FSG**S**SMNPA**R**SFGPAVAA	
OsTIP3;1		FDG**A**GMNPA**R**VFGPALVG	
OsTIP3;2		FDG**A**AMNPA**R**AFGPALVG	
OsTIP4;1		FSG**A**SMNPA**R**SFGPALAT	
OsTIP4;2		LTG**A**SMNPA**R**SFGPALAT	
OsTIP4;3		YSG**A**SMNPA**R**SFGPALAA	
OsTIP5;1		LTG**A**SMNPA**R**SFGPAVVS	
ZmTIP1;1		FDG**A**SMNPA**V**SFGPALVS	
ZmTIP1;2		FDG**A**SMNPA**V**SFGPAVVT	
ZmTIP2;1		FSG**G**SMNPA**R**SFGPAVAA	
ZmTIP2;2		FSG**G**SMNPA**R**SFGPAVAA	
ZmTIP2;3		FSG**G**SMNPA**R**SFGPAVAA	
ZmTIP3;1		FDG**A**GMNPA**R**VFGPALVG	
ZmTIP4;1		FTG**A**SMNPA**R**SFGPALAT	
ZmTIP4;2		FTG**A**SMNPA**R**SFGPAMAT	
ZmTIP4;3		LSG**A**SMNPA**R**SFGPAVAS	
ZmTIP4;4		FSG**A**SMNPA**R**SFGPALVA	
ZmTIP5;1		LTG**A**SMNPA**R**SFGPAVVS	
AtTIP1;1		FSG**A**SMNPA**V**AFGPAVVS	
AtTIP1;2		FSG**A**SMNPA**V**AFGPAVVS	
AtTIP1;3		FDG**A**SMNPA**V**SFGPAVVS	
AtTIP2;1		FSG**G**SMNPA**R**SFGPAVAA	
AtTIP2;2		FSG**G**SMNPA**R**SFGPAVVS	
AtTIP2;3		FSG**G**SMNPA**R**SFGPAVVS	
AtTIP3;1		FSG**A**SMNPA**R**AFGPALVG	
AtTIP3;2		FDG**A**SMNPA**R**AFGPALVG	
AtTIP4;1		FSG**A**SMNPA**R**SFGPALVS	
AtTIP5;1		FSG**G**SMNPA**C**AFGSAMVY	

LE NIP

1J4N	188	YTG**C**GINPA**R**SFGSSVIT	205
1FX8	197	LTG**F**AMNPA**R**DFGPKVFA	214
1RC2	180	VTN**T**SVNPA**R**STAVAI-F	196
1Z98	216	ITG**T**GINPA**R**SFGAAVIF	233
2B6O	178	YTG**A**GMNPA**R**SFAPAILT	195
2F2B	193	ISG**S**SLNPA**R**TFGPYLND	210
OsNIP1;1		ISG**A**SMNPA**R**SLGPAMIG	
OsNIP1;2		VTG**A**SMNPA**R**SLGPAMVA	
OsNIP1;3		ISG**A**SMNPA**R**TIGPAIIL	
OsNIP1;4		VSG**A**SMNPA**R**SIGPALVG	
OsNIP1;5		VTG**A**SMNPA**R**SLGPAMVA	
OsNIP2;1		ISG**G**SMNPA**R**TLGPALAS	
OsNIP2;2		VSG**G**SMNPA**R**TLAPAVAS	
OsNIP3;1		TTG**G**SMNPV**R**TLGPAVAA	
OsNIP3;2		STG**A**SMNPA**R**TLGPAIAT	
OsNIP3;3		STG**A**SMNPA**R**TIGAAIAT	
OsNIP3;4		STG**A**SMNPA**R**TLGTAIVA	
OsNIP3;5		STG**P**SMNPA**R**TIGAAVAT	
OsNIP4;1		VSG**G**SMNPA**R**TLGPAIVL	
ZmNIP1;1		VSG**A**SMNPA**R**SVGPALVS	
ZmNIP2;1		VSG**G**SMNPA**R**TLGPALAS	
ZmNIP2;2		VSG**G**SMNPA**R**TLAPAVAS	
ZmNIP3;1		TTG**G**SMNPV**R**TLGPAVAA	
AtNIP1;1		VSS**A**SMNPG**R**SLGPALVY	
AtNIP1;2		VSG**A**SMNPG**R**SLGPAMVY	
AtNIP2;1		VSG**A**SMNPA**R**SIGPALVW	
AtNIP3;1		ISG**A**SMNPA**R**SLGPALIW	
AtNIP4;1		ISG**A**SMNPA**R**SLGPALVM	
AtNIP4;2		ISG**A**SMNPA**R**SLGPAIVM	
AtNIP5;1		STG**G**SMNPV**R**TLGPAVAS	
AtNIP6;1		ATS**A**SMNPV**R**TLGPAIAA	
AtNIP7;1		ISG**G**SMNPA**R**SLGPAVVA	

LE SIP

1J4N	188	YTG**C**GINPA**R**SFGSSVIT	205
1FX8	197	LTG**F**AMNPA**R**DFGPKVFA	214
1RC2	180	VTN**T**SVNPA**R**STAVAI-F	196
1Z98	216	ITG**T**GINPA**R**SFGAAVIF	233
2B6O	178	YTG**A**GMNPA**R**SFAPAILT	195
2F2B	193	ISG**S**SLNPA**R**TFGPYLND	210
OsSIP1;1		YTG**P**SMNPA**N**AFGWAYVN	
OsSIP2;1		ITG**G**VMNPA**S**AFAWAYAR	
ZmSIP1;1		YTG**P**SMNPA**N**AFGWAYVN	
ZmSIP1;2		YTG**P**SMNPA**N**AFGWAYVN	
ZmSIP2;1		ITG**G**IMNPA**S**AFAWAYAR	
AtSIP1;1		YTG**P**AMNPA**I**AFGWAYMY	
AtSIP1;2		FTR**P**FMNPA**I**AFGWAYIY	
AtSIP2;1		LTG**G**CMNPA**A**VMGWAYAR	
